# Analysis of positional candidate genes in the AAA1 susceptibility locus for abdominal aortic aneurysms on chromosome 19

**DOI:** 10.1186/1471-2350-12-14

**Published:** 2011-01-19

**Authors:** John H Lillvis, Yoshiki Kyo, Gerard Tromp, Guy M Lenk, Ming Li, Qing Lu, Robert P Igo, Natzi Sakalihasan, Robert E Ferrell, Charles M Schworer, Zoran Gatalica, Susan Land, Helena Kuivaniemi

**Affiliations:** 1 Center for Molecular Medicine and Genetics, Wayne State University School of Medicine, Detroit, Michigan, USA; 2 Sigfried and Janet Weis Center for Research, Geisinger Clinic, Danville, 100 North Academy Avenue, Pennsylvania 17822-2610, USA; 3 Department of Epidemiology, Michigan State University, East Lansing, Michigan, USA; 4 Department of Epidemiology and Biostatistics, Case Western Reserve University, Cleveland, Ohio, USA; 5 Department of Cardiovascular Surgery, University Hospital of Liège, Liège, Belgium; 6 Department of Human Genetics, University of Pittsburgh, School of Public Health, Pittsburgh, Pennsylvania, USA; 7 Department of Pathology, Creighton University School of Medicine, Omaha, Nebraska, USA; 8 Applied Genomics Technology Center, Department of Obstetrics and Gynecology, Wayne State University School of Medicine, Detroit, Michigan, USA; 9 Department of Cardiovascular Surgery, Higashihiroshima Medical Center, Hiroshima, Japan; 10 Department of Human Genetics, University of Michigan, Ann Arbor, MI, USA

## Abstract

**Background:**

Abdominal aortic aneurysm (AAA) is a complex disorder with multiple genetic risk factors. Using affected relative pair linkage analysis, we previously identified an AAA susceptibility locus on chromosome 19q13. This locus has been designated as the AAA1 susceptibility locus in the Online Mendelian Inheritance in Man (OMIM) database.

**Methods:**

Nine candidate genes were selected from the AAA1 locus based on their function, as well as mRNA expression levels in the aorta. A sample of 394 cases and 419 controls was genotyped for 41 SNPs located in or around the selected nine candidate genes using the Illumina GoldenGate platform. Single marker and haplotype analyses were performed. Three genes (*CEBPG*, *PEPD *and *CD22*) were selected for DNA sequencing based on the association study results, and exonic regions were analyzed. Immunohistochemical staining of aortic tissue sections from AAA and control individuals was carried out for the CD22 and PEPD proteins with specific antibodies.

**Results:**

Several SNPs were nominally associated with AAA (p < 0.05). The SNPs with most significant p-values were located near the CCAAT enhancer binding protein (*CEBPG*), peptidase D (*PEPD*), and *CD22*. Haplotype analysis found a nominally associated 5-SNP haplotype in the *CEBPG*/*PEPD *locus, as well as a nominally associated 2-SNP haplotype in the *CD22 *locus. DNA sequencing of the coding regions revealed no variation in *CEBPG*. Seven sequence variants were identified in *PEPD*, including three not present in the NCBI SNP (dbSNP) database. Sequencing of all 14 exons of *CD22 *identified 20 sequence variants, five of which were in the coding region and six were in the 3'-untranslated region. Five variants were not present in dbSNP. Immunohistochemical staining for CD22 revealed protein expression in lymphocytes present in the aneurysmal aortic wall only and no detectable expression in control aorta. PEPD protein was expressed in fibroblasts and myofibroblasts in the media-adventitia border in both aneurysmal and non-aneurysmal tissue samples.

**Conclusions:**

Association testing of the functional positional candidate genes on the AAA1 locus on chromosome 19q13 demonstrated nominal association in three genes. *PEPD *and *CD22 *were considered the most promising candidate genes for altering AAA risk, based on gene function, association evidence, gene expression, and protein expression.

## Background

Abdominal aortic aneurysm (AAA) is an irreversible, progressive dilation of the abdominal aorta, occurring most frequently below the renal arteries. Known risk factors for AAA include smoking, male sex, comorbid vascular disease, increasing age, and family history [1]. AAA pathophysiology is complex, but fundamentally aneurysms arise from the loss of structural integrity and the consequent weakening of the vessel wall. Several extracellular matrix (ECM) degrading enzymes, including matrix metallopeptidases (MMPs) [2], cathepsins [3,4], and granzyme [5], have been implicated in the destruction and turnover of ECM proteins in the aortic wall. Infiltrating cells of the immune system are also found throughout the AAA wall [6] and are important to AAA development by producing ECM degrading enzymes and reactive oxygen species, as well as releasing proinflammatory cytokines that lead to further inflammation [7,8].

AAA also displays several characteristics consistent with being a multifactorial genetic disease [9]. Approximately 15% of AAA patients have a positive family history [10] and genetic association studies have identified risk alleles in cardiovascular risk genes such as *DAB2IP *[11], *CDKN2BAS *[12], *AGTR1 *[13,14], *ACE *[13,14], and *MTHFR *[14]. These polymorphisms, however, explain only a small fraction of an individual's genetic risk for AAA. A genome-wide DNA linkage analysis using an affected relative pair approach with covariates by our laboratory identified two linked chromosomal regions on 19q13 and 4q31 [15]. Linkage to chromosome 19q was replicated in a separate study in Dutch families using different statistical analyses [16]. Chromosome 19q13 may harbour risk alleles for several aneurysm phenotypes, as linkage to this region has also been reported for intracranial aneurysms [17-19] and the Kawasaki disease [20].

Several functional candidate genes reside within the chromosome 19 candidate interval. Peptidase D (*PEPD*; prolidase) is a manganese-dependent dipeptidyl peptidase that cleaves iminopeptides with C-terminal proline or hydroxyproline, which is the terminal step in collagen degradation [21]. The chromosome 19q candidate interval also contains several genes that may be relevant to immune system function in AAA. *CD22 *is a sialic-acid binding protein expressed in B-lymphocytes, which can inhibit signalling through the B-cell receptor and therefore alter B-cell function [22]. B-cells are found in the AAA wall and *CD22 *has been implicated in autoimmune pathologies characterized by similar inflammatory and ECM changes seen in some AAAs [23].

Here we report on an analysis of candidate genes selected from the chromosome 19q13 linkage interval. Nine functional positional candidate genes (*PEPD*, *CEBPA*, *CEBPG*, *GPI*, *HAMP*, *CD22*, *NFKBID*, *TYROBP*, and *HCST*) were identified as having annotated function in either the immune system or in ECM turnover. Fifty-five SNPs covering the major haplotype blocks in each gene were selected. Two genes with evidence of association, *CD22 *and *PEPD*, were investigated further by exon sequencing and immunohistochemical staining of aneurysmal and non-aneurysmal aortic tissue samples.

## Methods

### Human samples for genotyping

AAA cases used in genotyping (n = 394; 79% male) were collected at the University of Pittsburgh, the University of Liége in Liége, Belgium, and Dalhousie University Hospital in Nova Scotia, Canada. Canadian controls were spouses of AAA cases. Belgian controls were patients with diagnoses other than AAA and treated at the same hospital as the AAA cases. Details on the recruitment have been published previously and the samples have been used in prior genetic association studies [12,24,25]. The collection of samples was approved by the Institutional Review Board of Wayne State University and each recruitment site.

### DNA isolation and whole genome amplification (WGA)

Genomic DNA used in genotyping and sequencing was isolated from peripheral blood as described previously [25]. A total of 10 ng of genomic DNA was amplified by strand-displacement amplification [26] with Phi29 polymerase using the Genomiphi Kit (GE Healthcare Biosciences, Piscataway, NJ) according to the manufacturer's instructions. Amplified DNA was diluted 1:100 for use in genotyping and DNA sequencing reactions.

### RNA isolation and cDNA synthesis

Total RNA was isolated from cultured skin fibroblasts as previously described [27]. cDNA was prepared from 250 ng of RNA using SuperScript III First-Strand Synthesis Supermix (Invitrogen, Carlsbad, CA). Residual RNA was removed by RNAse H treatment.

### Genetic association study

Genes in the linkage interval on chromosome 19 were prioritized functionally using Gene Ontology (GO) [28] and Kyoto Encyclopedia of Genes and Genomes (KEGG) [29] annotations. mRNA expression of each gene in AAA and control abdominal aorta was also assessed using data from a previously described microarray-based mRNA expression profiling study [30]. Altogether 55 SNPs with a minor allele frequency (MAF) of 0.10 or greater that were representative of major haplotype blocks in the Caucasian population were identified in and around the nine positional functional candidate genes selected for the study. Each SNP was assessed for its SNP quality score from Illumina and a custom Illumina BeadChip was designed using those SNPs considered to have sufficient designability. The SNPs are listed in Additional file 1, Table S1.

Power calculations were performed using the Genetic Power Calculator [31] (http://pngu.mgh.harvard.edu/~purcell/gpc/). We assumed that the polymorphism and the disease locus were in complete linkage disequilibrium (LD) and that they had the same allele frequencies, i.e., the polymorphism was the disease locus. Assuming a disease locus with an additive effect and a disease prevalence of 0.02, our sample size of 394 cases and 419 controls had an 80% power to detect a susceptibility locus with a genotypic relative risk (GRR) ≥ 1.2 (2.4 for two copies of risk alleles) at a significance level of 0.05 for a SNP with a high risk allele frequency (HAF) ≥ 0.2.

SNPs were genotyped using the Illumina GoldenGate assay [32] and the call rate of each SNP was evaluated using GenCall Software (version 6.1.3.28, Illumina, San Diego, CA). Deviation from Hardy-Weinberg equilibrium (HWE) was evaluated using an exact test [33] as implemented in Haploview [34] and nominal p-values without correction for multiple testing are reported in Additional File 1. Allelic association was tested using a Pearson χ^2 ^test with one degree of freedom (DF). To assess the potential for population stratification, HWE and allelic association were also tested separately in the Belgian and Canadian subpopulations. An additional test of association was performed using the general linear mixed model approach implemented in the Statistical Analysis for Genetic Epidemiology (S.A.G.E.) [35] program ASSOC, which yields both Wald and likelihood ratio test (LRT) statistics. Both tests gave very similar results and therefore, we report only results from the LRT which is considered more robust in small sample sizes. False discovery rates (FDR) are reported for the LRT. Haplotype association was analyzed using a score test [36] implemented in the haplo.score function in the package HaploStats for the R statistical language and environment [35]. For genes with evidence of association, LD plots were generated from the case and control genotypes separately using HaploView to examine LD structure.

### DNA sequencing of functional positional candidate genes

Genomic DNA and RNA samples used in DNA sequencing are listed in Additional files 2 (Table S2) and 3 (Table S3). Primer pairs to amplify the coding sequence of *CEBPG*, cDNA sequence of *PEPD*, and exonic sequences of *CD22 *were designed using PrimerQuest^SM^, a primer design tool based on Primer3 software [37] available on the Integrated DNA Technologies website (http://www.idtdna.com/Scitools/Applications/Primerquest/; IDT, Coralville, IA). Primers were selected using predicted melting points, primer hairpins and primer-dimer interactions calculated using the SciTools programs in PrimerQuest^SM^. DNA amplification by PCR was performed under the following conditions. Final concentrations were 50 mM KCl, 1.5 or 2 mM MgCl_2 _(primer-pair specific; see Additional file 4, Table S4), 2 μM dNTPs, 0.167 μM of each primer, 0.025 U/μl AmpliTaq Gold enzyme (Applied Biosystems) in 10 mM Tris-HCl, pH 8.3. Template DNA constituted 1/10^th ^of the final volume for WGA-genomic DNA or 1/20^th ^for cDNA. Template was denatured by heating to 94°C for 10 minutes, which was followed by 40 cycles of denaturation (94°C), annealing (variable) and elongation (72°C), and a final three-minute elongation incubation at 72°C. Detailed cycle times and annealing temperatures are found in Additional file 4, Table S4.

PCR products were separated by agarose gel electrophoresis to assess the sizes of bands and the specificity of the reaction. PCR products with single clean bands of the correct molecular weight were purified for DNA sequencing using Montage™ PCR purification columns (Millipore, Billerica, MA).

DNA sequencing was performed using the Cycle Sequencing (Applied Biosystems, Foster City, CA) method at the Applied Genomics Technology Center (AGTC) at Wayne State University. Primers used for DNA sequencing can be found in Additional file 5, Table S5. Sequence variants were identified by alignment of sequences using BioX software (available at https://www.lagercrantz.name/projects/biox) and manual examination of the sequence plots.

### Functional analysis of sequence variants

To assess the potential function of identified sequence variants, *in silico *functional analyses were carried out. The SIFT (Sorting Intolerant from Tolerant) algorithm [38] was used to predict the structural effects of non-synonymous substitutions. NetPhos [39] was used to predict the creation or destruction of phosphorylation sites. To assess the potential effect of 3'-untranslated region (UTR) sequence variants on microRNA (miRNA) binding, predicted binding sites were obtained from the MicroCosm Targets Database [40,41] (http://www.ebi.ac.uk/enright-srv/microcosm/htdocs/targets/v5/).

### Immunohistochemical staining to analyze protein expression in human aortic tissues

Control non-aneurysmal aortas (n = 7) were obtained post-mortem at autopsy. Exclusion criteria were known malignancy or systemic infection. AAA tissues (n = 9) were obtained from elective surgical repair operations when the aneurysmal sac was trimmed as part of surgery. Tissue samples were removed solely for the purpose of graft placement and would otherwise be discarded. Tonsil (for CD22) or kidney (for PEPD) tissue were used as positive controls. Nonspecific IgG in lieu of primary antibody served as a negative control. All tissue samples were fixed in buffered formalin solution, routinely processed and then embedded in paraffin. Details on aortic samples used can be found in Additional file 6: Table S6.

Immunohistochemical staining was carried out using 5 μm sections of formalin-fixed paraffin embedded aortic tissue. Sections were dried in an oven and deparaffinized using sequential rinses in Histoclear xylene substitute (National Diagnostics U.S.A., Atlanta, GA), 100% ethanol, 95% ethanol and water. Heat-induced antigen retrieval was performed by microwave-treatment of slides submerged in citrate buffer. Endogenous peroxidase was blocked by placing slides in 3% methanol/peroxide block solution for 10 min. The slides were incubated with primary antibody directed against PEPD (1:250 dilution; catalog number 12218-1-AP, Proteintech Group, Inc., Chicago, IL) or CD22 (1:100 dilution; catalog number ab953, Abcam Inc., Cambridge, MA) on an automatic immunostainer (Autostainer, DAKO, Carpinteria, CA). A secondary antibody with avidin-biotin peroxidase amplification from DAKO was used and the signal detected using diaminobenzidine as a chromogen.

## Results

### Selection of functional candidate genes on AAA1 locus

**Figure 1:**
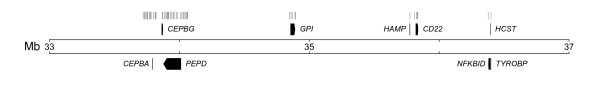
**Map of relative positions of candidate genes in the AAA1 linkage interval**. The relative positions of the nine candidate genes studied are shown above (plus strand) or below (minus strand) the black scale bars depending on the direction of transcription. The positions of genotyped SNPs are indicated at the top as gray lines.

The AAA1 linkage interval (Figure 1) spans approximately 4 Mbp from 33 Mbp to 37 Mbp (NCBI build 37.3) on chromosome 19q13 and contains over 100 genes. In order to identify a smaller set of strong candidate genes for further study, GO and KEGG functional annotations were used to identify nine genes with functions relevant to AAA pathogenesis (Table 1). Eight of these genes were selected based on an annotated function in the immune system, as immune involvement in AAA pathogenesis has been well studied [7]. Additionally, *PEPD*, was identified as a candidate gene based on its activity in collagen turnover and potential for contributing to ECM remodelling in the AAA wall [21].

**Table 1:** Functional positional candidate genes studied by genetic association

Symbol	GO Category	KEGG Pathway	SNP (N)	AAA Exp	Cont Exp	**Diff Score**^**1**^
*PEPD*	NA	NA	25	1300	957	10.52
*CEBPG*	immune response; others	NA	2	222	252	-3.27
*CEBPA*	myeloid cell differentiation	NA	16	630	118	31.32^2^
*GPI*	humoral immune response	NA	5	1136	759	11.57
*HAMP*	immune response; others	NA	1	136	ND	13.57
*CD22*	immune response; others	B-cell receptor signaling pathway	4	578	ND	16.17
*NFKBID*	inflammatory response	NA	1	22.4	ND	23.80^2^
*TYROBP*	cellular defense response	natural killer cell mediated cytotoxicity	1	4973	801	41.15^2^
*HCST*	NA	natural killer cell mediated cytotoxicity	0	736	148	33.44^2^

Nine genes were identified within the AAA linkage intervals on chromosome 19 (AAA1 locus) with annotated GO or KEGG functions relevant to AAA. mRNA expression levels for each gene in both AAA and control abdominal aortic tissue, as well as a score indicating the differences in expression levels (Diff score), were obtained from our previously published work, details of which can be found in [30] and the microarray data can be obtained at the Gene Expression Omnibus (GEO) database (Series# GSE7084; http://www.ncbi.nlm.nih.gov/geo/).

^1^The Diff Score is generated using a custom algorithm from Illumina. A Diff Score of 13 corresponds to an approximate p value of 0.05.

^2^Significantly different between AAA and control tissues after correction for multiple testing (FDR <0.05).

Chr, chromosome; NA, not applicable; ND, not detected; Exp, Expression; Cont, Control

Since genes not expressed in aortic tissue, either diseased or non-diseased, would be less likely to contribute to aneurysm formation, mRNA expression of each candidate gene was assessed using microarray expression profiles of AAA and control aortic tissue. As all of the genes selected by functional annotation were expressed in either AAA or control abdominal aorta, no genes were excluded by this criterion. Furthermore, four of these genes, *CEBPA*, *NFKBID*, *HCST *and *TYROBP*, had significantly higher expression in AAA tissue than in age-, sex-, and ethnicity-matched control aortas and *CD22 *was expressed only in AAA tissue (Table 1).

### Candidate gene association study

Fifty-five SNPs in or around the nine functional candidate genes found in the AAA1 locus were genotyped. Eleven SNPs were excluded from further analysis since they were either monomorphic or had low genotype call rates (Additional file 1, Table S1). This number can be considered high, but is expected in a custom array where several of the SNP assays had not been previously validated. Additionally, the excluded SNPs were located in *CEPBA *and *PEPD*, where the genotyping coverage was excellent even without including the failed assays. Testing for deviation from HWE was performed in the remaining 44 SNPs and four SNPs (rs7248389, rs752237, rs736289 and rs4239576) that showed deviation were identified (Additional file 1, Table S1). One of the four SNPs also showed evidence of association and since deviation from HWE can result from association, this SNP (rs7248389) was included in the final analysis. Three of the four SNPs did not show evidence of association, and were subsequently excluded (rs752237, rs736289 and rs4239576) from further analysis leaving 41 SNPs for the final analyses. In the cases of these three variants the deviation from HWE was likely due to technical problems in genotyping.

Using an allelic χ^2 ^test of association, eight SNPs in three genes, *CD22 *(2 SNPs), *PEPD *(5 SNPs), and *HAMP *(1 SNP), were identified as being nominally associated with AAA (p < 0.05; Table 2). When tested for genotypic association using logistic regression, six of these SNPs were nominally associated, two were not associated, and an additional SNP in *GPI *was identified (Table 2). Although not significantly associated, SNPs in *NFKBID *did approach nominal significance (p < 0.10) by this test. In Table 2 we also present the FDR-values for the LRT statistics. Only the association with rs7248389 in *PEPD *remained significant when correcting for multiple testing.

**Table 2:** Genetic association study results for SNPs with nominal p < 0.05.

SNP	Gene	Alleles (Minor/Major)	MAF (Cases; Controls) **χ**^**2 **^**test P-value**			LRT	
			
			Combined	Canadian	Belgian	P-value	FDR
rs7248389	*PEPD*	T/C	0.395; 0.478	0.404; 0.493	0.379; 0.469		
			**0.0009**	**0.0220**	**0.0084**	**0.001**	**0.037**
rs889140^1^	*PEPD*	C/T	0.326; 0.395	0.328; 0.411	0.320; 0.386		
			**0.0043**	**0.0248**	0.0513	**0.0025**	0.088
rs2267574	*CD22*	A/T	0.105; 0.147	0.106; 0.180	0.110; 0.129		
			**0.0112**	**0.0047**	0.4185	**0.016**	0.15
rs2241380	*PEPD*	G/A	0.419; 0.362	0.436; 0.371	0.404; 0.356		
			**0.0184**	0.0831	0.1536	**0.035**	0.185
rs756796	*CD22*	A/G	0.280; 0.230	0.273; 0.222	0.289; 0.234		
			**0.02**	0.1179	0.068	**0.016**	0.164
rs7250833	*PEPD*	T/C	0.332; 0.282	0.328; 0.278	0.337; 0.285		
			**0.0317**	0.1582	0.0987	**0.043**	0.219
rs7251432	*HAMP*	A/G	0.511; 0.459	0.520; 0.437	0.506; 0.472		
			**0.0361**	**0.0296**	0.5193	0.050	0.210
rs11880064	*PEPD*	C/T	0.415; 0.364	0.431; 0.371	0.402; 0.360		
			**0.0367**	0.1045	0.2124	0.059	0.190
rs580391	*GPI*	A/C	0.203; 0.243	0.196; 0.245	0.218; 0.243		
			0.0519	0.1143	0.4012	**0.038**	0.237

Tests for allelic association were carried out by a χ^2 ^test for each SNP. P-values < 0.05 are shown in bold. The genotypic test of association was performed using the general linear mixed model approach implemented in the Statistical Analysis for Genetic Epidemiology (S.A.G.E.) [35] program ASSOC, which yields the likelihood ratio test (LRT) and the Wald statistics. Only LRT results are reported here since both LRT and Wald gave similar results. SNPs are ranked here by χ^2 ^test p-value in the combined population. In the alleles column minor allele is listed first based on the results seen in controls. For rs7251432 the minor allele was different for cases than controls.

^1^SNP was out of Hardy-Weinberg equilibrium in Belgian controls.

Since there were multiple associated SNPs in *PEPD *and *CD22*, the LD structure of the genotyped SNPs in each gene was examined using LD plots (Additional file 7, Figure S1 and Additional file 8, Figure S2).

To identify population-specific effects, allelic association was evaluated in the Canadian and Belgian subpopulations separately (Table 2). For a majority of the SNPs identified above, evidence of association was stronger in the Canadian subpopulation. Four SNPs (rs2267574, rs7248389, rs889140, and rs7251432) were nominally associated in the Canadian subpopulation, but only one of these (rs7248389) showed evidence for association in the Belgian subpopulation. One associated SNP located in *PEPD *(rs7248389) showed nominal deviation from HWE. All nominally associated SNPs and their p-values for the different tests are summarized in Table 2.

Haplotypic tests of association were also performed on SNPs in *CD22 *and *PEPD*. Using a sliding window of five consecutive SNPs, nominally significant haplotypic association (p = 0.0026) was identified in the region of *PEPD *at the SNPs rs10500265-rs6510383-rs7248389-rs7250833-rs2241380 (Table 3). Nominally significant haplotypic association (p = 0.0065) was also identified in two SNPs in CD22 (rs756796-rs2267574) (Table 3).

**Table 3:** Significantly associated haplotypes in *PEPD *and *CD22 *genes

Gene and SNPs in the haplotype	Alleles	Haplotype	Haplotype			
			
			Frequency in cases	Frequency in controls	OR [95% CI]	P-value
*PEPD*						
		[CTTCA]	0.328	0.367	Reference	
rs10500265	C/G	[CTCCA]	0.136	0.154	1.049 [0.761, 1.445]	
rs6510383	C/T	[CTTCG]	0.066	0.068	1.062 [0.687, 1.644]	
rs7248389	C/T	[CCCCA]	0.115	0.096	1.422 [0.997, 2.028]	0.0026
rs7250833	C/T	[CTCCG]	0.021	0.013	1.872 [0.745, 4.706]	
rs2241380	A/G	[GCCTG]	0.095	0.075	1.512 [1.020, 2.242]	
		[CTCTG]	0.226	0.188	1.435 [1.087, 1.894]	
*CD22*						
rs756796	A/G	[GA]	0.105	0.147	0.716 [0.528,0.971]	
rs2267574	A/T	[GT]	0.615	0.623	Reference	0.0065
		[AT]	0.280	0.230	1.253 [0.995, 1.577]	

The most common haplotype was used as a reference (OR of 1) and the other haplotypes were compared to that.

### DNA sequencing of exons in candidate genes with putative association

Three genes, *CD22*, *CEBPG*, and *PEPD*, were chosen for DNA sequencing. No significantly associated SNPs were detected within *CEBPG*, but it was chosen for sequencing since it is in close proximity to *PEPD *and could plausibly contribute to the association signal detected in that gene. *CD22 *was also considered an intriguing candidate gene since it was expressed in AAA tissue and its expression was not detectable in non-aneurysmal abdominal aorta.

Since the functional consequences associated with exon sequence changes are often easier to predict than with intronic sequences, exon sequencing covering a minimum of the protein coding regions of each gene was performed. The protein coding region of *CEPBG *was sequenced from the genomic DNA of 21 AAA cases, 10 familial and 11 sporadic, and two controls. *CEBPG *has a large 3'-UTR that we chose not to sequence because of the relatively weak evidence for association within the gene. No sequence variants were identified within the regions sequenced and therefore no further analysis was pursued with this gene.

**Figure 2:**
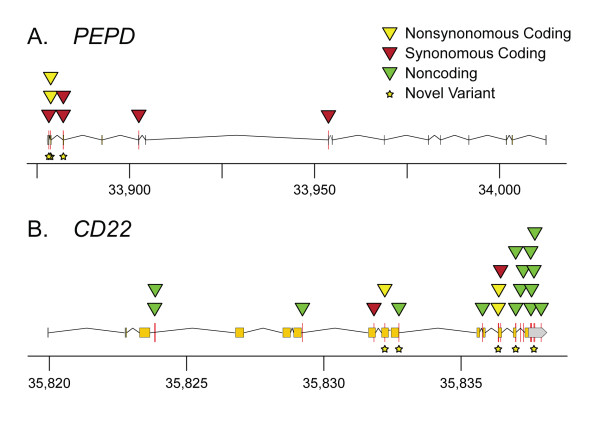
**Sequence variants identified by candidate gene sequencing**. Variants in *PEPD *(A) and *CD22 *(B) plotted to show their relative chromosomal positions and positions relative to gene features. Exons are indicated by the boxes above the x-axis. *PEPD *is transcribed right to left (minus strand) and *CD22 *is transcribed left to right (plus strand). Chromosomal coordinates are in kilobase pairs (kb).

*PEPD *contains 15 small exons that are spaced over a large genomic distance. cDNA was used as a template for sequencing since the spliced transcript could be covered in only three PCR reactions. Seven sequence variants were identified in *PEPD *when 21 AAA cases and two controls were analyzed (Table 4; Figure 2A; Additional file 9, Table S7; Additional file 10, Table S8). Six individuals showed no variation in their *PEPD *sequence, 12 were heterozygous for one variant, four were heterozygous for two variants and one individual was homozygous for the minor allele of one variant (Additional file 9, Table S7). Four of the seven variants were present in only 1/23 (1/46 alleles) individuals, one was present in 3/23 (3/46 alleles), one in 5/23 (5/46 alleles) and one in 9/23 (10/46 alleles) (Additional file 9, Table S7).

**Table 4:** Sequence changes detected by cDNA sequencing of *PEPD*

	**Chr. Pos. (Build 37)**^**1**^	Gene Feature	Allele Change	SNP Identifier	**MAF (Caucasian)**^**2**^	mRNA Position	Amino Acid	**Residue Change**^**3**^
1	33,953,912	Exon 9	T- > C	rs3745969	0.063	793	220	Y- > Y
2	33,902,652	Exon 11	T- > C	rs74988985	0.021	877	248	G- > G
3	33,882,255	Exon 13	C- > T		0.021	1231	366	H- > H
4	33,882,222	Exon 13	C- > T	rs17569	0.104 (0.155)	1264	377	H- > H
5	33,878,845	Exon 14	G- > A		0.021	1427	432	A- > T
6	33,878,837	Exon 14	C- > T	rs17570	0.229 (0.246)	1436	435	L- > F
7	33,878,340	Exon 15	G- > T		0.021	1525	464	L- > L

^1^Chromosomal position obtained from the National Center for Biotechnology Information (NCBI).

^2^MAF calculated for each SNP from sequencing data on 23 individuals presented in the current study. MAFs in parentheses are from HapMap CEU or other Caucasian population available at the NCBI website.

^3^Single letter amino acids codes are used in Residue Change column.

Chr. Pos., Chromosomal position; MAF, minor allele frequency.

For details, see Additional file 9, Table S7 and Additional file 10, Table S8.

Three of the *PEPD *sequence variants identified were novel, in that they were not in either the dbSNP or Celera databases. Two SNPs coded for non-synonymous amino acid changes: rs17570, which results in a leucine to phenylalanine substitution at amino acid position 435, and a novel variant resulting in an alanine to threonine substitution at amino acid 432 (Table 4).

*CD22 *has 14 exons and was sequenced from genomic DNA because closer exon spacing allowed for multiple exons to be covered in a single reaction. A total of 20 sequence variants were identified, including five novel variants when analyzing 22 AAA cases and 3 controls (Table 5; Figure 2B; Additional file 11, Table S9; Additional file 12, Table S10). Five variants were in the protein-coding region, including three coding for non-synonymous amino acid substitutions, and six were found within the 3'-UTR. All but one of the 25 individuals were heterozygous for at least one sequence variant. Two individuals showed a high degree of variability and were heterozygous for seven variants (Additional file 11: Table S9).

**Table 5:** Sequence changes detected by *CD22 *exon sequencing

	Chr. Pos. (Build 37)	Gene Feature	Allele Change	SNP Identifier	MAF (Caucasian)	mRNA Position	Amino Acid	Residue Change
1	35,823,999	Intron 3	C- > T	rs881456	0.021 (0.000)			
2	35,824,019	Intron 3	C- > G	rs10419538	0.104 (0.181)			
3	35,829,381	Intron 6	A- > G	rs7248108	0.042 (0.042)			
4	35,831,986	Exon 7	C- > T	rs25677	0.042 (0.000)	1529	484	C- > C
5	35,832,381	Exon 8	G- > A		0.021	1721	548	R- > K
6	35,832,886	Intron 9	T- > A		0.021			
7	35,835,940	Intron 10	C- > T	rs45453699	0.313			
8	35,836,512	Exon 12	A- > G		0.021	2293	739	R- > G
9	35,836,530	Exon 12	G- > A	rs10406069	0.104 (0.292)	2311	745	G- > D
10	35,836,600	Exon 12	C- > A	rs34826052	0.042	2381	768	P- > P
11	35,837,148	Intron 13	G- > A	hCV25603572	0.021 (0.05)			
12	35,837,150	Intron 13	C- > T		0.021			
13	35,837,327	Intron 13	C- > T	rs58156121	0.063			
14	35,837,428	Intron 13	G- > A	rs12985354	0.208 (0.383)			
15	35,837,694	3'-UTR	C- > T	rs73031792	0.250	2715		
16	35,837,705	3'-UTR	A- > G	rs35529786	0.167	2726		
17	35,837,724-5	3'-UTR	GC I/D^1^	rs34472317	0.042	2745-6		
18	35,837,813	3'-UTR	C- > T		0.042	2834		
19	35,837,846	3'-UTR	C- > T	rs16970255	0.042 (0.000)	2867		
20	35,838,076	3'-UTR	C- > T	rs3088063	0.042 (0.055)	3097		

^1^Variant allele is GC insertion.

For definitions, see footnote to Table 4.

For details, see Additional file 11, Table S9 and Additional file 12, Table S10.

Three of the sequence variants identified in the *CD22 *were non-synonymous (R > K, R > G and G > D) amino acid changes (Table 5). Two of the three were very rare (MAF = 0.02), whereas one (rs10406069) had a MAF = 0.104. We tested this variant for genetic association in our case-control set, but found no differences in the allele or genotype frequencies between cases and controls.

### Predicted functional changes

Several *in silico *analyses were used to predict whether any of the observed sequence changes might alter protein function or regulation of gene expression. To assess the potential effect of non-synonymous amino acid substitutions, each sequence variant was analyzed with the SIFT algorithm, which predicts whether amino acid changes will not be tolerated. SIFT predicted that all five amino acid substitutions coded by the sequence variants identified in *CD22 *and *PEPD *would be tolerated. Structural modeling of *PEPD *demonstrated that the identified amino acid substitutions A432T and L435F, resided in close proximity in a region that appeared to tolerate structural changes (not shown).

The 3'-UTR of many genes acts as a binding site for miRNAs in post-transcriptional gene regulation [42]. To find out if any of the six sequence variants identified in the 3'-UTR of *CD22 *might interrupt miRNA binding, predicted miRNA binding sites were obtained from MicroCosm database. We found no evidence that the variants changed the binding sites of any miRNAs (not shown).

### CD22 and PEPD proteins are expressed in aortic wall

**Figure 3:**
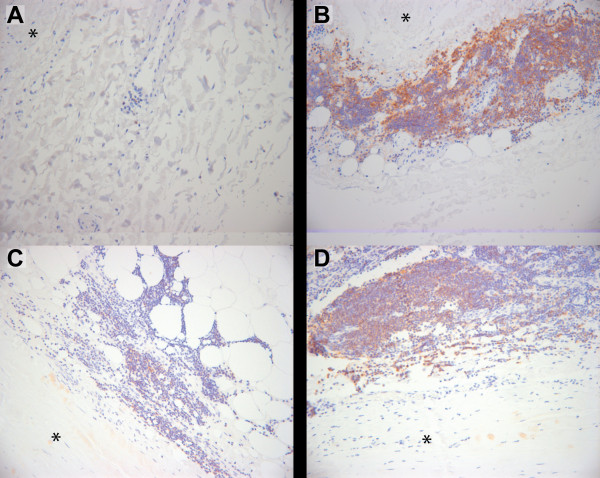
**Immunohistochemical staining of CD22 demonstrates expression in AAA tissue associated lymphocytes**. Immunohistochemical staining using a commercially available specific antibody against CD22 was performed on formalin-fixed paraffin embedded tissue sections of non-aneurysmal abdominal aorta (A) and AAA (B, C, D). Negative control staining with non-immune serum showed no staining (data not shown). Images are centered on lymphocytes (small round cells with sparse cytoplasm) primarily seen in the adventitial layer. The adjacent media is indicated with an asterisk. Positive staining appears reddish-brown with hematoxylin counterstaining in blue.

**Figure 4:**
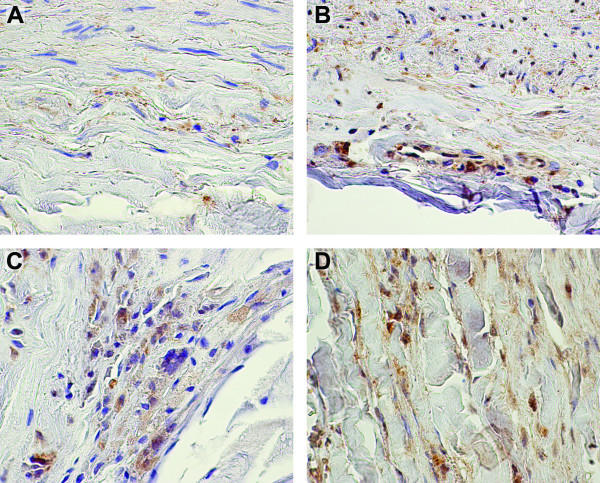
**Immunohistochemical staining of PEPD demonstrates expression in both aneurysmal and non-aneurysmal aortic tissue**. Immunohistochemical staining using a commercially available specific antibody against PEPD was performed on formalin-fixed paraffin embedded tissue sections of non-aneurysmal abdominal aorta (A, B) and AAA (C, D) with staining observed in medial (A) and adventitial layers of controls and throughout the aneurysmal wall. Negative control staining with non-immune serum showed no staining (data not shown). Regions of positive staining appear reddish-brown with hematoxylin counterstaining in blue.

Protein expression of *CD22 *and *PEPD *in the aorta was confirmed by immunohistochemical staining of paraffin-embedded tissue sections of AAA and control abdominal aorta (Figures 3 and 4). Staining demonstrated expression of PEPD protein in both AAA and control sections; expression was observed in fibroblasts and myofibroblasts in the media-adventitia border in both aneurysmal and non-aneurysmal tissue samples. Consistent with previous observations [43-45], staining for CD22 protein showed an expansion of lymphocytes in AAA tissue as compared with non-aneurysmal aorta.

## Discussion

Strong evidence of linkage exists on chromosome 19 in the AAA1 locus [15,16], but no specific risk loci have yet been discovered. We identified nine functional positional candidate genes within this interval for further study by genetic association. Two of these, *PEPD *and *CD22*, showed evidence of nominal association in multiple SNPs, although only one SNP remained significant after correction for multiple testing. Additionally, significant five- and two-SNP haplotypes were identified for *PEPD *and *CD22*, respectively.

Evidence of association in *PEPD *and *CD22*, was of considerable interest. Based on gene function, gene expression, and protein expression, these genes are plausible candidates for contributing to the risk of developing an AAA. The role of PEPD in collagen metabolism makes it a strong functional candidate for AAA. Although it is unlikely that complete loss of PEPD function alters AAA risk, given that it results in the severe phenotype of prolidase deficiency, polymorphisms that alter PEPD activity could contribute to AAA formation, particularly if they resulted in increased enzymatic activity [46]. Increased PEPD activity has been associated with increased collagen production, both in *in vitro *experiments [21,47] and in keloid scars [48]. Excess collagen production is observed in AAA tissue [49], and has been suggested to contribute to weakening of the aneurysm wall [50]. PEPD is also regulated by several mechanisms relevant to AAA pathogenesis. Nitric oxide, which may contribute to AAA formation [51], upregulates PEPD activity [47]. Similar to other metalloproteases, PEPD activity is sensitive to doxycycline [52], a tetracycline antibiotic with anti-inflammatory properties that have been studied for their therapeutic potential in AAA [53-56]. Estrogen can regulate PEPD *in vitro *[57,58], an interesting observation given that AAA is six times more common in men than women [1]. Finally, increased serum activity of PEPD has been associated with diseases such as asthma [59], coronary artery disease [60], and fatty liver disease [61].

CD22 is a sialic-acid binding protein found on cells of B-cell lineage. It can inhibit signaling through the B-cell receptor [22], suggesting that impairment of gene function might lead to dysregulation of the immune response and either autoimmune disease or increased inflammation. Supporting this hypothesis is evidence of a role for CD22 in systemic sclerosis; anti-CD22 autoantibodies contribute to systemic sclerosis pathogenesis [62] and there is evidence of association between a SNP in *CD22 *and limited cutaneous systemic sclerosis [23]. Systemic sclerosis is characterized by immune infiltration and excessive fibrosis, both of which are observed in AAA subtype known as inflammatory AAA [63]. Given that patients with inflammatory AAA are more likely to have a positive family history [64], *CD22 *polymorphisms could contribute to AAA risk. On the other hand, *Cd22 *knockout mice display a minimal phenotype with no evidence of increased autoimmune disease [22]. It is plausible, however, that polymorphisms in *CD22 *could modulate the immune response in an inflammatory disease such as AAA through changes in the amount of CD22 protein produced in a cell or an alteration of the gene's function in B-cell signalling.

Exon sequencing of *PEPD *and *CD22 *showed that both have polymorphic coding regions. Polymorphisms that did not result in amino acid substitution or changes to the 3'-UTR were not considered further, since their functional effects are difficult to predict. After investigation for predicted functional consequences, all of the sequence changes identified in the coding regions or 3'-UTR of *CD22 *and *PEPD *appeared to be tolerated.

One limitation of this study was that the sample size used for genetic association provided only modest power. Although we did observe nominal association in several genes, only one remained significant after correction for multiple testing. Our results should, however, be considered in light of the prior evidence of linkage on chromosome 19q13. While this evidence does indicate the presence of variants contributing to AAA risk at this locus, the differences between linkage and association do not make identification of associated variants a given. Linkage is tolerant of allelic heterogeneity, whereas a variant must be sufficiently common to be detected by association. Therefore, a biologically relevant variant in a linked region may exhibit either weak association, as we observed, or no evidence of association (extensive allelic heterogeneity). Furthermore, if there is also locus heterogeneity in genetic risk factors between populations, increasing sample size using subjects from a different population than that originally studied may actually decrease the evidence of association observed. In light of this and our observation of association at multiple SNPs for each gene, we consider our findings for *PEPD *and *CD22 *worth additional studies.

It is possible that the associations seen here resulted from spurious associations or from population stratification. A recent association study examining SNPs from the chromosome 19 linkage interval in the Dutch population found several nominally associated SNPs but none remained significant for multiple testing [65]. Interestingly, the SNP showing the strongest evidence of association resided in *CEPBG*, which is in agreement with our observation of association in the region of *CEBPG *and *PEPD *(Figure 1).

Finally, by focusing on functional candidate genes rather than genotyping the entire linkage region, it is possible that linked and associated genes were overlooked. The strategy presented here was based on reducing the number of tests due to the limited power of our sample and the increased ability to interpret associations in genes of known function. Recent technological improvements in high-throughput sequencing provide a potentially more efficient approach to follow-up of linkage results [66,67]. Deep sequencing at high coverage of linkage intervals in members of linked families has identified novel loci. For late age-at-onset diseases the lack of complete nuclear pedigrees and inability to identify definitively unaffected individuals, will reduce the approach to affected relative pair sequencing.

## Conclusions

In the present study nine functional positional candidate genes on AAA1 locus on chromosome 19 were investigated. Two of the genes, *CD22 *and *PEPD *showed modest level of evidence of being involved in AAA pathogenesis. This evidence came from a nominal association of SNPs residing in these genes to AAA, identification of novel sequence changes and expression of these proteins in aneurysmal tissue. If replicated in independent studies, the findings provide important information about AAA pathogenesis.

## Competing interests

The authors declare that they have no competing interests.

## Authors' contributions

JHL participated in the design of the study, prepared templates for DNA sequencing, carried out *in silico *analysis, and drafted the manuscript. YK and GML catalogued phenotypic information, processed samples, designed the genotyping experiments, and carried out quality control for genotyping data. GT participated in the design of the study, isolated genomic DNA and RNA, participated in the analysis of genotyping and sequencing data, and interpreted results. ML, QL and RPI carried out the statistical analyses of the genotyping data. NS and REF recruited patients and controls for the genetic association study. CMS and ZG carried out and interpreted the immunohistochemical analyses. SL supervised the genotyping and DNA sequencing. HK obtained funding for the study, participated in the design of the study, isolated genomic DNA and RNA, participated in the analysis of genotyping and sequencing data, interpreted results, and participated in drafting the manuscript. All authors read and approved the final manuscript.

## Pre-publication history

The pre-publication history for this paper can be accessed here:

http://www.biomedcentral.com/1471-2350/12/14/prepub

## Supplementary Material

Additional File 1 **Table S1. List of all 55 SNPs chosen for the study**. Each SNP genotyped for this study ordered by chromosomal coordinate. SNP RefSeq number, gene symbol, minor allele, chromosome 19 coordinate, p-value for HWE test, MAF in the entire population and for Canadian and Belgian populations separately (cases and controls separately), p-values for the allelic test for combined analysis as well as for Canadian and Belgian population separately, and LRT p-values are provided in tabular format. RefSeq numbers contain hyperlinks to the NCBI site. Click here for file

Additional File 2 **Table S2. Genomic DNA samples used for sequencing of *CEBPG *and *CD22***. For each sample used in sequencing, case/control status, nationality of origin, sex and sequencing status of *CD22 *and *CEBPG *provided in tabular format. Click here for file

Additional File 3 **Table S3. cDNA samples used for sequencing of *PEPD***. Case/control status, nationality of origin, sex and family history of each individual whose sample was used in the sequencing of *PEPD *provided in tabular format. Click here for file

Additional File 4 **Table S4. PCR primer pairs for *CEPBG, PEPD *and *CD22***. For each PCR reaction, a description of each reaction, primer pair sequences and orientation, product sizes, annealing temperatures, and the cycling times and magnesium chloride concentrations used provided in tabular format. Click here for file

Additional File 5 **Table S5. Sequencing primers used for *CEBPG*, *PEPD *and *CD22***. For each sequencing reaction, a description of the reaction, the primer sequences and orientation, and predicted melting points for primer provided in tabular format. Click here for file

Additional File 6 **Table S6. Human tissue samples used in immunohistochemical analysis of CD22 and PEPD**. Donor age, sex, case/control status and control cause of death if known. Click here for file

Additional File 7 **Figure S1. Linkage disequilibrium (LD) plots of genotyped SNPs in *CEBPG *and *PEPD *for cases (A) and controls (B) separately**. LD at the *CEBPG*/*PEPD *locus plotted separately for cases and controls using r^2 ^as the statistic. Approximate locations of genes and SNPs were plotted along the x-axis above plots. Nominally associated SNPs are indicated with an asterisk. Click here for file

Additional File 8 **Figure S2. LD plots of genotyped SNPs in *CD22 *for cases (A) and controls (B) separately**. LD at the *CD22 *locus plotted separately for cases and controls using r^2 ^as the statistic. Approximate location of *CD22 *and SNPs were plotted along the x-axis above plots. Nominally associated SNPs are indicated with an asterisk. Click here for file

Additional File 9 **Table S7. *PEPD *sequence changes by sample number**. Table showing genotype at each sequence variant detected by sequencing in each individual sequenced. Sample numbers refer to Additional file 3, Table S3, and sequence change number refers to Table 4. Click here for file

Additional File 10 **Table S8. Flanking sequences of the *PEPD *sequence variants**. Gene feature, 15 bp flanking sequences and SNP identifier, if available, for each sequence variant. Click here for file

Additional File 11 **Table S9. *CD22 *sequence changes by sample number**. Table showing genotype at each sequence variant detected by sequencing in each individual sequenced. Sample numbers refer to Additional file 2, Table S2, and sequence change number refers to Table 5. Click here for file

Additional File 12 **Table S10. Flanking sequences of the *CD22 *sequence variants**. Gene feature, 15 bp flanking sequences and SNP identifier, if available, for each sequence variant. Click here for file

## References

[B1] Lederle FA Johnson GR Wilson SE Chute EP Hye RJ Makaroun MS Barone GW Bandyk D Moneta GL Makhoul RG The aneurysm detection and management study screening program: validation cohort and final results. Aneurysm Detection and Management Veterans Affairs Cooperative Study Investigators Arch Intern Med 2000 160 1425 1430 10.1001/archinte.160.10.1425 10826454

[B2] Keeling WB Armstrong PA Stone PA Bandyk DF Shames ML An overview of matrix metalloproteinases in the pathogenesis and treatment of abdominal aortic aneurysms Vasc Endovascular Surg 2005 39 457 464 10.1177/153857440503900601 16382266

[B3] Liu J Sukhova GK Yang JT Sun J Ma L Ren A Xu WH Fu H Dolganov GM Hu C Cathepsin L expression and regulation in human abdominal aortic aneurysm, atherosclerosis, and vascular cells Atherosclerosis 2006 184 302 311 10.1016/j.atherosclerosis.2005.05.012 15982660

[B4] Sukhova GK Shi GP Simon DI Chapman HA Libby P Expression of the elastolytic cathepsins S and K in human atheroma and regulation of their production in smooth muscle cells J Clin Invest 1998 102 576 583 10.1172/JCI181 9691094 PMC508918

[B5] Chamberlain CM Ang LS Boivin WA Cooper DM Williams SJ Zhao H Hendel A Folkesson M Swedenborg J Allard MF Perforin-independent extracellular granzyme B activity contributes to abdominal aortic aneurysm Am J Pathol 2010 176 1038 1049 10.2353/ajpath.2010.090700 20035050 PMC2808106

[B6] Koch AE Haines GK Rizzo RJ Radosevich JA Pope RM Robinson PG Pearce WH Human abdominal aortic aneurysms. Immunophenotypic analysis suggesting an immune-mediated response Am J Pathol 1990 137 1199 1213 1700620 PMC1877681

[B7] Kuivaniemi H Platsoucas CD Tilson MD Aortic aneurysms: an immune disease with a strong genetic component Circulation 2008 117 242 252 10.1161/CIRCULATIONAHA.107.690982 18195185 PMC3001294

[B8] Nischan J Lenk GM Boddy AM Lillvis JH Tromp G Kuivaniemi H Laurent A, Morel E. Hauppage Abdominal aortic aneurysms- a complex genetic disease Aneurysms: Types, Risks, Formation and Treatment 2009 NY: Nova Science Publishers, Inc

[B9] Hinterseher I Tromp G Kuivaniemi H Genes and Abdominal Aortic Aneurysm Ann Vasc Surg 2010 in press 21146954 10.1016/j.avsg.2010.09.004 PMC3058859

[B10] Lillvis JH Lenk GM Kuivaniemi H Upchurch G, Criado E Genetics of Abdominal Aortic Aneurysms Aortic Aneurysms: Pathogenesis and Treatment 2008 Totowa, NJ: Humana Press Inc 1 26

[B11] Gretarsdottir S Baas AF Thorleifsson G Holm H den Heijer M de Vries JP Kranendonk SE Zeebregts CJ van Sterkenburg SM Geelkerken RH Genome-wide association study identifies a sequence variant within the DAB2IP gene conferring susceptibility to abdominal aortic aneurysm Nat Genet 2010 42 692 697 10.1038/ng.622 20622881 PMC4157066

[B12] Helgadottir A Thorleifsson G Magnusson KP Grétarsdottir S Steinthorsdottir V Manolescu A Jones GT Rinkel GJ Blankensteijn JD Ronkainen A The same sequence variant on 9p21 associates with myocardial infarction, abdominal aortic aneurysm and intracranial aneurysm Nat Genet 2008 40 217 224 10.1038/ng.72 18176561

[B13] Jones GT Thompson AR van Bockxmeer FM Hafez H Cooper JA Golledge J Humphries SE Norman PE van Rij AM Angiotensin II type 1 receptor 1166C polymorphism is associated with abdominal aortic aneurysm in three independent cohorts Arterioscler Thromb Vasc Biol 2008 28 764 770 10.1161/ATVBAHA.107.155564 18239157 PMC2775049

[B14] McColgan P Peck GE Greenhalgh RM Sharma P The genetics of abdominal aortic aneurysms: a comprehensive meta-analysis involving eight candidate genes in over 16,700 patients Int Surg 2009 94 350 358 20302034

[B15] Shibamura H Olson JM van Vlijmen-Van Keulen C Buxbaum SG Dudek DM Tromp G Ogata T Skunca M Sakalihasan N Pals G Genome scan for familial abdominal aortic aneurysm using sex and family history as covariates suggests genetic heterogeneity and identifies linkage to chromosome 19q13 Circulation 2004 109 2103 2108 10.1161/01.CIR.0000127857.77161.A1 15096456

[B16] van Vlijmen-van Keulen CJ Rauwerda JA Pals G Genome-wide linkage in three Dutch families maps a locus for abdominal aortic aneurysms to chromosome 19q13.3 Eur J Vasc Endovasc Surg 2005 30 29 35 10.1016/j.ejvs.2004.12.029 15933979

[B17] Olson JM Vongpunsawad S Kuivaniemi H Ronkainen A Hernesniemi J Ryynanen M Kim LL Tromp G Search for intracranial aneurysm susceptibility gene(s) using Finnish families BMC Med Genet 2002 3 7 10.1186/1471-2350-3-7 12153705 PMC119849

[B18] van der Voet M Olson JM Kuivaniemi H Dudek DM Skunca M Ronkainen A Niemela M Jaaskelainen J Hernesniemi J Helin K Intracranial aneurysms in Finnish families: confirmation of linkage and refinement of the interval to chromosome 19q13.3 Am J Hum Genet 2004 74 564 571 10.1086/382285 14872410 PMC1182270

[B19] Yamada S Utsunomiya M Inoue K Nozaki K Inoue S Takenaka K Hashimoto N Koizumi A Genome-wide scan for Japanese familial intracranial aneurysms: linkage to several chromosomal regions Circulation 2004 110 3727 3733 10.1161/01.CIR.0000143077.23367.18 15569837

[B20] Onouchi Y Tamari M Takahashi A Tsunoda T Yashiro M Nakamura Y Yanagawa H Wakui K Fukushima Y Kawasaki T Hata A A genomewide linkage analysis of Kawasaki disease: evidence for linkage to chromosome 12 J Hum Genet 2007 52 179 190 10.1007/s10038-006-0092-3 17160344

[B21] Surazynski A Miltyk W Palka J Phang JM Prolidase-dependent regulation of collagen biosynthesis Amino Acids 2008 35 731 738 10.1007/s00726-008-0051-8 18320291

[B22] Nitschke L CD22 and Siglec-G: B-cell inhibitory receptors with distinct functions Immunol Rev 2009 230 128 143 10.1111/j.1600-065X.2009.00801.x 19594633

[B23] Hitomi Y Tsuchiya N Hasegawa M Fujimoto M Takehara K Tokunaga K Sato S Association of CD22 gene polymorphism with susceptibility to limited cutaneous systemic sclerosis Tissue Antigens 2007 69 242 249 10.1111/j.1399-0039.2007.00801.x 17493148

[B24] Ogata T Gregoire L Goddard KA Skunca M Tromp G Lancaster WD Parrado AR Lu Q Shibamura H Sakalihasan N Evidence for association between the HLA-DQA locus and abdominal aortic aneurysms in the Belgian population: a case control study BMC Med Genet 2006 7 67 10.1186/1471-2350-7-67 16879749 PMC1559600

[B25] Ogata T Shibamura H Tromp G Sinha M Goddard KA Sakalihasan N Limet R MacKean GL Arthur C Sueda T Genetic analysis of polymorphisms in biologically relevant candidate genes in patients with abdominal aortic aneurysms J Vasc Surg 2005 41 1036 1042 10.1016/j.jvs.2005.02.020 15944607 PMC1249499

[B26] Dean FB Hosono S Fang L Wu X Faruqi AF Bray-Ward P Sun Z Zong Q Du Y Du J Comprehensive human genome amplification using multiple displacement amplification Proc Natl Acad Sci USA 2002 99 5261 5266 10.1073/pnas.082089499 11959976 PMC122757

[B27] Tromp G Wu Y Prockop DJ Madhatheri SL Kleinert C Earley JJ Zhuang J Norrgard O Darling RC Abbott WM Sequencing of cDNA from 50 unrelated patients reveals that mutations in the triple-helical domain of type III procollagen are an infrequent cause of aortic aneurysms J Clin Invest 1993 91 2539 2545 10.1172/JCI116490 8514866 PMC443315

[B28] Ashburner M Ball CA Blake JA Botstein D Butler H Cherry JM Davis AP Dolinski K Dwight SS Eppig JT Gene ontology: tool for the unification of biology. The Gene Ontology Consortium Nat Genet 2000 25 25 29 10.1038/75556 10802651 PMC3037419

[B29] Kanehisa M Goto S KEGG: kyoto encyclopedia of genes and genomes Nucleic Acids Res 2000 28 27 30 10.1093/nar/28.1.27 10592173 PMC102409

[B30] Lenk GM Tromp G Weinsheimer S Gatalica Z Berguer R Kuivaniemi H Whole genome expression profiling reveals a significant role for immune function in human abdominal aortic aneurysms BMC Genomics 2007 8 237 10.1186/1471-2164-8-237 17634102 PMC1934369

[B31] Purcell S Cherny SS Sham PC Genetic Power Calculator: design of linkage and association genetic mapping studies of complex traits Bioinformatics 2003 19 149 150 10.1093/bioinformatics/19.1.149 12499305

[B32] Steemers FJ Gunderson KL Illumina, Inc Pharmacogenomics 2005 6 777 782 10.2217/14622416.6.7.777 16207153

[B33] Wigginton JE Cutler DJ Abecasis GR A note on exact tests of Hardy-Weinberg equilibrium Am J Hum Genet 2005 76 887 893 10.1086/429864 15789306 PMC1199378

[B34] Barrett JC Fry B Maller J Daly MJ Haploview: analysis and visualization of LD and haplotype maps Bioinformatics 2005 21 263 265 10.1093/bioinformatics/bth457 15297300

[B35] S.A.G.E Statistical Analysis for Genetic Epidemiology 2009 Cleveland: Department of Epidemiology and Biostatistics, Case Western Reserve University Release 6.0.1 edition

[B36] Schaid DJ Rowland CM Tines DE Jacobson RM Poland GA Score tests for association between traits and haplotypes when linkage phase is ambiguous Am J Hum Genet 2002 70 425 434 10.1086/338688 11791212 PMC384917

[B37] Rozen S Skaletsky H Primer3 on the www for general users and for biologist programmers Methods Mol Biol 2000 132 365 386 10547847 10.1385/1-59259-192-2:365

[B38] Ng PC Henikoff S SIFT: Predicting amino acid changes that affect protein function Nucleic Acids Res 2003 31 3812 3814 10.1093/nar/gkg509 12824425 PMC168916

[B39] Blom N Gammeltoft S Brunak S Sequence and structure-based prediction of eukaryotic protein phosphorylation sites J Mol Biol 1999 294 1351 1362 10.1006/jmbi.1999.3310 10600390

[B40] Griffiths-Jones S Grocock RJ van Dongen S Bateman A Enright AJ miRBase: microRNA sequences, targets and gene nomenclature Nucleic Acids Res 2006 34 D140 144 10.1093/nar/gkj112 16381832 PMC1347474

[B41] Griffiths-Jones S Saini HK van Dongen S Enright AJ miRBase: tools for microRNA genomics Nucleic Acids Res 2008 36 D154 158 10.1093/nar/gkm952 17991681 PMC2238936

[B42] Bartel DP MicroRNAs: genomics, biogenesis, mechanism, and function Cell 2004 116 281 297 10.1016/S0092-8674(04)00045-5 14744438

[B43] Pearce WH Koch AE Cellular components and features of immune response in abdominal aortic aneurysms Ann N Y Acad Sci 1996 800 175 185 10.1111/j.1749-6632.1996.tb33308.x 8958992

[B44] Stella A Gargiulo M Pasquinelli G Preda P Faggioli GL Cenacchi G D'Addato M The cellular component in the parietal infiltrate of inflammatory abdominal aortic aneurysms (IAAA) Eur J Vasc Surg 1991 5 65 70 10.1016/S0950-821X(05)80929-6 2009988

[B45] Walton LJ Powell JT Parums DV Unrestricted usage of immunoglobulin heavy chain genes in B cells infiltrating the wall of atherosclerotic abdominal aortic aneurysms Atherosclerosis 1997 135 65 71 10.1016/S0021-9150(97)00152-4 9395274

[B46] Lupi A Tenni R Rossi A Cetta G Forlino A Human prolidase and prolidase deficiency: an overview on the characterization of the enzyme involved in proline recycling and on the effects of its mutations Amino Acids 2008 35 739 752 10.1007/s00726-008-0055-4 18340504

[B47] Surazynski A Liu Y Miltyk W Phang JM Nitric oxide regulates prolidase activity by serine/threonine phosphorylation J Cell Biochem 2005 96 1086 1094 10.1002/jcb.20631 16167338

[B48] Duong HS Zhang QZ Le AD Kelly AP Kamdar R Messadi DV Elevated prolidase activity in keloids: correlation with type I collagen turnover Br J Dermatol 2006 154 820 828 10.1111/j.1365-2133.2006.07167.x 16634881

[B49] Baxter BT Davis VA Minion DJ Wang YP Lynch TG McManus BM Abdominal aortic aneurysms are associated with altered matrix proteins of the nonaneurysmal aortic segments J Vasc Surg 1994 19 797 802 8170033 10.1016/s0741-5214(94)70004-4

[B50] Deguchi JO Huang H Libby P Aikawa E Whittaker P Sylvan J Lee RT Aikawa M Genetically engineered resistance for MMP collagenases promotes abdominal aortic aneurysm formation in mice infused with angiotensin II Lab Invest 2009 89 315 326 10.1038/labinvest.2008.167 19153555 PMC2932654

[B51] Lizarbe TR Tarin C Gomez M Lavin B Aracil E Orte LM Zaragoza C Nitric oxide induces the progression of abdominal aortic aneurysms through the matrix metalloproteinase inducer EMMPRIN Am J Pathol 2009 175 1421 1430 10.2353/ajpath.2009.080845 19779140 PMC2751539

[B52] Karna E Palka J Wolczynski S Doxycycline-induced inhibition of prolidase activity in human skin fibroblasts and its involvement in impaired collagen biosynthesis Eur J Pharmacol 2001 430 25 31 10.1016/S0014-2999(01)01372-3 11698059

[B53] Abdul-Hussien H Hanemaaijer R Verheijen JH van Bockel JH Geelkerken RH Lindeman JH Doxycycline therapy for abdominal aneurysm: Improved proteolytic balance through reduced neutrophil content J Vasc Surg 2009 49 741 749 10.1016/j.jvs.2008.09.055 19268776

[B54] Baxter BT Pearce WH Waltke EA Littooy FN Hallett JW Jr Kent KC Upchurch GR Jr Chaikof EL Mills JL Fleckten B Prolonged administration of doxycycline in patients with small asymptomatic abdominal aortic aneurysms: report of a prospective (Phase II) multicenter study J Vasc Surg 2002 36 1 12 10.1067/mva.2002.125018 12096249

[B55] Curci JA Mao D Bohner DG Allen BT Rubin BG Reilly JM Sicard GA Thompson RW Preoperative treatment with doxycycline reduces aortic wall expression and activation of matrix metalloproteinases in patients with abdominal aortic aneurysms J Vasc Surg 2000 31 325 342 10.1016/S0741-5214(00)90163-0 10664501

[B56] Lindeman JH Abdul-Hussien H van Bockel JH Wolterbeek R Kleemann R Clinical trial of doxycycline for matrix metalloproteinase-9 inhibition in patients with an abdominal aneurysm: doxycycline selectively depletes aortic wall neutrophils and cytotoxic T cells Circulation 2009 119 2209 2216 10.1161/CIRCULATIONAHA.108.806505 19364980

[B57] Miltyk W Anchim T Wolczynski S Palka J Estrogen-dependent regulation of prolidase activity in breast cancer MCF-7 cells Gynecol Endocrinol 1999 13 166 174 10.3109/09513599909167551 10451808

[B58] Wolczynski S Surazynski A Swiatecka J Palka J Estrogenic and antiestrogenic effects of raloxifene on collagen metabolism in breast cancer MCF-7 cells Gynecol Endocrinol 2001 15 225 233 11447735

[B59] Cakmak A Zeyrek D Atas A Celik H Aksoy N Erel O Serum prolidase activity and oxidative status in patients with bronchial asthma J Clin Lab Anal 2009 23 132 138 10.1002/jcla.20303 19288447 PMC6649124

[B60] Yildiz A Demirbag R Yilmaz R Gur M Altiparmak IH Akyol S Aksoy N Ocak AR Erel O The association of serum prolidase activity with the presence and severity of coronary artery disease Coron Artery Dis 2008 19 319 325 10.1097/MCA.0b013e32830042ba 18607169

[B61] Kayadibi H Gultepe M Yasar B Ince AT Ozcan O Ipcioglu OM Kurdas OO Bolat B Benek YZ Guveli H Diagnostic value of serum prolidase enzyme activity to predict the liver histological lesions in non-alcoholic fatty liver disease: a surrogate marker to distinguish steatohepatitis from simple steatosis Dig Dis Sci 2009 54 1764 1771 10.1007/s10620-008-0535-0 18989777

[B62] Odaka M Hasegawa M Hamaguchi Y Ishiura N Kumada S Matsushita T Komura K Sato S Takehara K Fujimoto M Autoantibody-mediated regulation of B cell responses by functional anti-CD22 autoantibodies in patients with systemic sclerosis Clin Exp Immunol 2010 159 176 184 10.1111/j.1365-2249.2009.04059.x 19919568 PMC2810386

[B63] Tang T Boyle JR Dixon AK Varty K Inflammatory abdominal aortic aneurysms Eur J Vasc Endovasc Surg 2005 29 353 362 15749035 10.1016/j.ejvs.2004.12.009

[B64] Nitecki SS Hallett JW Jr Stanson AW Ilstrup DM Bower TC Cherry KJ Jr Gloviczki P Pairolero PC Inflammatory abdominal aortic aneurysms: a case-control study J Vasc Surg 1996 23 860 868 10.1016/S0741-5214(96)70249-5 8667508

[B65] Baas AF Medic J van't Slot R de Vries JP van Sambeek MR Geelkerken BH Boll BP Grobbee DE Wijmenga C Ruigrok YM Blankensteijn JD Association Study of Single Nucleotide Polymorphisms on Chromosome 19q13 With Abdominal Aortic Aneurysm Angiology 2010 61 243 247 10.1177/0003319709354752 20156811

[B66] Rehman AU Morell RJ Belyantseva IA Khan SY Boger ET Shahzad M Ahmed ZM Riazuddin S Khan SN Friedman TB Targeted capture and next-generation sequencing identifies C9orf75, encoding taperin, as the mutated gene in nonsyndromic deafness DFNB79 Am J Hum Genet 2010 86 378 388 10.1016/j.ajhg.2010.01.030 20170899 PMC2833391

[B67] Rosa-Rosa JM Gracia-Aznarez FJ Hodges E Pita G Rooks M Xuan Z Bhattacharjee A Brizuela L Silva JM Hannon GJ Benitez J Deep sequencing of target linkage assay-identified regions in familial breast cancer: methods, analysis pipeline and troubleshooting PLoS One 2010 5 e9976 10.1371/journal.pone.0009976 20368986 PMC2848842

