# Supplementary material for: Analysis of positional candidate genes in the AAA1 susceptibility locus for abdominal aortic aneurysms on chromosome 19
Source: BMC Med Genet. 2011 Jan 19;12:14. doi: 10.1186/1471-2350-12-14 (PMC3037298; doi:10.1186/1471-2350-12-14)
Supplement: Additional File 1 — Table S1. List of all 55 SNPs chosen for the study. Each SNP genotyped for this study ordered by chromosomal coordinate. SNP RefSeq number, gene symbol, minor allele, chromosome 19 coordinate, p-value for HWE test, MAF in the entire population and for Canadian and Belgian populations separately (cases and controls separately), p-values for the allelic test for combined analysis as well as for Canadian and Belgian population separately, and LRT p-values are provided in tabular format. RefSeq numbers contain hyperlinks to the NCBI site. [file 1471-2350-12-14-S1.PDF]

Additional File 1:

Table S1. List of all 55 SNPs used in the study. The chromosome 19q13 candidate interval for AAA (AAA1 locus) identified in a DNA linkage study (Shibamura et al. 2004) was searched for putative candidate genes. SNPs were then selected in or around these genes.

					Minor Allele Frequency						P value			
				P-value	Combined		Canadian		Belgian		χ^2 (allele test)			LRT
rs number	Gene	Minor Allele	Coordinate[1]	HWE[2]	Case	Control	Case	Control	Case	Control	Combined	Canad.	Belgian	Combined
rs10410558 [3]	*CEBPA*	G	33,725,857											
rs752237 [4]	*CEBPA*	A	33,731,800	0.049	0.135	0.147	0.149	0.168	0.119	0.136	0.463	0.505	0.464	0.37
rs748680	*CEBPA*	T	33,733,928	0.44	0.082	0.079	0.079	0.097	0.089	0.069	0.821	0.416	0.286	0.69
rs7256230 [3]	*CEBPA*	T	33,742,370											
rs8107162	*CEBPA*	A	33,744,872	0.95	0.427	0.405	0.436	0.411	0.419	0.401	0.353	0.494	0.596	0.19
rs4432367 [3]	*CEBPA*	C	33,752,920											
rs736289 [4]	*CEBPA*	C	33,757,062	0.0013	0.377	0.409	0.380	0.384	0.366	0.424	0.183	0.909	0.091	0.077
rs8100802 [3]	*CEBPA*	A	33,768,921											
rs1990966	*CEBPA*	A	33,775,326	1	0.370	0.357	0.360	0.364	0.399	0.353	0.584	0.914	0.165	0.67
rs1423056	*CEBPA*	T	33,780,444	0.92	0.344	0.315	0.327	0.334	0.365	0.304	0.214	0.830	0.058	0.26
rs877567	*CEBPA*	A	33,782,911	1	0.092	0.085	0.106	0.077	0.078	0.089	0.605	0.196	0.569	0.33
rs7251505 [3]	*CEBPA*	A	33,802,542											
rs8100151	*CEBPA*	G	33,804,399	0.084	0.161	0.153	0.145	0.157	0.174	0.151	0.658	0.657	0.358	0.6
rs3848600	*CEBPA*	A	33,815,485	0.34	0.221	0.208	0.241	0.200	0.206	0.213	0.544	0.192	0.823	0.41
rs12608723	*CEBPA*	G	33,817,133	0.25	0.123	0.153	0.131	0.116	0.104	0.174	0.082	0.543	0.004	0.13
rs7253865 [3]	*CEBPA*	G	33,821,248											
rs1469084	*CEBPG*	A	33,868,013	0.80	0.432	0.475	0.441	0.497	0.431	0.458	0.089	0.098	0.437	0.06
rs3745968	*CEBPG*	G	33,872,410	0.96	0.117	0.104	0.145	0.103	0.087	0.104	0.419	0.098	0.400	0.34
rs7258031	*PEPD*	T	33,886,354	1	0.321	0.346	0.324	0.315	0.317	0.364	0.284	0.785	0.153	0.49
rs889140	*PEPD*	C	33,889,000	0.13	0.326	0.395	0.328	0.411	0.320	0.386	0.004	0.025	0.051	0.002
rs731839 [3]	*PEPD*	T	33,899,065											
rs4805034 [3]	*PEPD*	G	33,905,985											
rs1005731	*PEPD*	C	33,908,717	0.99	0.263	0.233	0.297	0.207	0.224	0.248	0.160	0.007	0.409	0.058
rs10500265	*PEPD*	G	33,914,285	0.78	0.100	0.091	0.125	0.086	0.076	0.093	0.535	0.099	0.362	0.34
rs4239576 [4]	*PEPD*	G	33,915,360	0.0019	0.336	0.357	0.331	0.348	0.330	0.362	0.371	0.640	0.343	0.55
rs6510383	*PEPD*	C	33,918,787	0.92	0.222	0.202	0.244	0.162	0.202	0.224	0.322	0.008	0.447	0.18
rs7248389	*PEPD*	T	33,932,315	0.020	0.395	0.478	0.404	0.493	0.379	0.469	0.0009	0.022	0.008	0.00076
rs7250833	*PEPD*	T	33,937,277	0.25	0.332	0.282	0.328	0.278	0.337	0.285	0.032	0.158	0.099	0.042
rs2241380	*PEPD*	G	33,951,126	0.21	0.419	0.362	0.436	0.371	0.404	0.356	0.018	0.083	0.154	0.034
rs3745970	*PEPD*	A	33,954,773	0.58	0.098	0.087	0.125	0.079	0.072	0.091	0.441	0.051	0.316	0.27
rs11880064	*PEPD*	C	33,964,181	0.17	0.415	0.364	0.431	0.371	0.402	0.360	0.037	0.105	0.212	0.058

rs10425678	*PEPD*	T	33,977,396	0.097	0.474	0.498	0.461	0.470	0.485	0.479	0.346	0.069	0.871	0.31
rs3786915	*PEPD*	G	33,987,424	0.59	0.360	0.318	0.352	0.295	0.363	0.331	0.077	0.107	0.332	0.069
rs33840 [3]	*PEPD*	A	34,010,289											
rs33847	*PEPD*	A	34,020,401	0.30	0.129	0.152	0.131	0.152	0.124	0.152	0.191	0.409	0.257	0.31
rs4805903	*PEPD*	A	34,028,391	0.48	0.304	0.308	0.288	0.268	0.327	0.330	0.893	0.573	0.935	0.95
rs153703	*PEPD*	A	34,029,698	0.37	0.282	0.291	0.304	0.315	0.257	0.277	0.696	0.761	0.515	0.79
rs996088 [3]	*PEPD*	A	34,037,282											
rs2278260	*PEPD*	T	34,048,308	0.58	0.488	0.495	0.490	0.493	0.465	0.496	0.512	0.931	0.265	0.6
rs1529486	*PEPD*	C	34,055,581	0.87	0.405	0.422	0.429	0.430	0.383	0.418	0.480	0.967	0.305	0.55
rs891035	*PEPD*	C	34,060,938	0.28	0.286	0.294	0.292	0.305	0.294	0.288	0.718	0.718	0.868	0.81
rs11672393 [3]	*PEPD*	C	34,064,936											
rs12327733	*PEPD*	G	34,065,833	0.55	0.285	0.283	0.283	0.267	0.275	0.292	0.922	0.626	0.573	0.76
rs580391	*GPI*	A	34,848,251	0.85	0.203	0.243	0.196	0.245	0.218	0.243	0.052	0.114	0.401	0.038
rs2099099	*GPI*	T	34,859,457	0.85	0.404	0.414	0.399	0.450	0.410	0.393	0.688	0.171	0.604	0.6
rs2278266	*GPI*	A	34,870,256	0.61	0.192	0.174	0.193	0.200	0.187	0.159	0.349	0.819	0.282	0.24
rs2301263	*GPI*	G	34,890,322	0.31	0.192	0.173	0.194	0.190	0.185	0.164	0.339	0.904	0.425	0.25
rs1559195	*GPI*	T	34,894,820	0.39	0.071	0.073	0.071	0.089	0.065	0.063	0.893	0.370	0.905	0.65
rs7251432	*HAMP*	A [5]	35,775,441	0.65	0.489	0.459	0.480	0.437	0.494	0.472	0.036	0.030	0.519	0.05
rs756796	*CD22*	A	35,809,800	0.92	0.280	0.230	0.273	0.222	0.289	0.234	0.020	0.118	0.068	0.016
rs2267574	*CD22*	A	35,825,143	1	0.105	0.147	0.106	0.180	0.110	0.129	0.011	0.005		0.016
rs2239511	*CD22*	A	35,831,537	0.45	0.151	0.183	0.148	0.205	0.157	0.170	0.087	0.045	0.618	0.19
rs3746250	*CD22*	G	35,835,582	0.46	0.324	0.293	0.329	0.312	0.306	0.282	0.169	0.634	0.432	0.098
rs11880466	*NFKBID*	C	36,377,975	0.28	0.218	0.258	0.202	0.238	0.241	0.269	0.061	0.245	0.365	0.054
rs3817624	*TYROBP*	T	36,398,899	0.59	0.121	0.092	0.116	0.107	0.121	0.084	0.063	0.711	0.069	0.21

[1] Genomic coordinates were obtained from NCBI (build 37.3)
[2] P values for HWE in controls; nominal significance, not corrected for multiple testing.
[3] The SNP did not pass quality control, or was monomorphic in the populations studied here.
[4] The SNP both deviated from HWE in controls and showed no evidence for association, and was removed from the final analyses.
[5] The minor allele for cases is G, rather than A; controls agree with HapMap in that A is the minor allele.

More detailed information about each SNP is available at NCBI through a hyperlink by clicking on the rs-number on the far left column.

Shibamura H, Olson JM, van Vlijmen-van Keulen C, Buxbaum SG, Dudek DM, Tromp G, Ogata T, Skunca M, Sakalihasan N, Pals G, Limet R, MacKean GL, Defawe O, Verloes A, Arthur C, Lossing AG, Burnett M, Sueda T, Kuivaniemi H: **Genome scan for familial abdominal aortic aneurysm using sex and family history as covariates suggests genetic heterogeneity and identifies linkage to chromosome 19q13.** *Circulation* 2004, 109: 2103-2108.
