# Supplementary material for: Analysis of positional candidate genes in the AAA1 susceptibility locus for abdominal aortic aneurysms on chromosome 19
Source: BMC Med Genet. 2011 Jan 19;12:14. doi: 10.1186/1471-2350-12-14 (PMC3037298; doi:10.1186/1471-2350-12-14)
Supplement: Additional File 2 — Table S2. Genomic DNA samples used for sequencing of CEBPG and CD22. For each sample used in sequencing, case/control status, nationality of origin, sex and sequencing status of CD22 and CEBPG provided in tabular format. [file 1471-2350-12-14-S2.PDF]

**Additional File 2:**

**Table S2. Genomic DNA samples used for sequencing of *CEBPG* and *CD22*.**

| Sample | Status | Origin | Sex | *CEBPG* | *CD22* |
|---|---|---|---|---|---|
| 1 | Case[1] | The Netherlands | M | Yes | Yes |
| 2 | Case[1] | USA | F | Yes | Yes |
| 3 | Case[1] | Canada | F | Yes | Yes |
| 4 | Case[1] | Canada | M | Yes | Yes |
| 6 | Case[1] | Belgium | M | Yes | Yes |
| 7 | Case[1] | Canada | M | Yes | Yes |
| 8 | Case[1] | The Netherlands | M | Yes | Yes |
| 9 | Case[1] | USA | M | Yes | Yes |
| 10 | Case[1] | UK | M | Yes | Yes |
| 11 | Case[1] | USA | F | Yes | Yes |
| 12 | Case | UK | F | Yes | Yes |
| 13 | Case | Canada | F | Yes | Yes |
| 14 | Case | Canada | F | Yes | Yes |
| 15 | Case | Canada | M | Yes | Yes |
| 16 | Case | Canada | M | Yes | Yes |
| 17 | Case | Canada | M | Yes | Yes |
| 18 | Case | Canada | F | Yes | Yes |
| 19 | Case | The Netherlands | M | Yes | Yes |
| 20 | Case | The Netherlands | M | Yes | Yes |
| 21 | Case | Belgium | M | Yes | Yes |
| 22 | Case | UK | F | Yes | Yes |
| 23 | Case | USA | F | No | Yes |
| 24 | Control | USA | F | Yes | Yes |
| 25 | Control | USA | F | No | Yes |
| 27 | Control | Canada | F | No | Yes |
| 28 | Control | Canada | M | No | Yes |

Genomic DNA was isolated from the blood of AAA cases and controls and underwent whole-genome amplification for use in PCR amplification. All AAA cases had a positive family history for AAA (Kuivaniemi et al. 2003). Controls were unrelated individuals married to the AAA cases.

[1]Case belongs to a family that contributed to the linkage signal on chromosome 19 (Shibamura et al. 2004). Cases sequenced here are unrelated to each other.

Kuivaniemi H, Shibamura H, Arthur C, Berguer R, Cole CW, Juvonen T, Kline RA, Limet R, MacKean G, Norrgård Ö, Pals G, Powell JT, Rainio P, Sakalihasan N, van Vlijmen-van Keulen C, Verloes A, Tromp G. **Familial abdominal aortic aneurysms: Collection of 233 multiplex families.** *J Vasc Surg* 2003, 37: 340-345.

Shibamura H, Olson JM, van Vlijmen-van Keulen C, Buxbaum SG, Dudek DM, Tromp G, Ogata T, Skunca M, Sakalihasan N, Pals G, Limet R, MacKean GL, Defawe O, Verloes A, Arthur C, Lossing AG, Burnett M, Sueda T, Kuivaniemi H: **Genome scan for familial abdominal aortic aneurysm using sex and family history as covariates suggests genetic heterogeneity and identifies linkage to chromosome 19q13.** *Circulation* 2004, 109: 2103-2108.
