# Supplementary material for: Analysis of positional candidate genes in the AAA1 susceptibility locus for abdominal aortic aneurysms on chromosome 19
Source: BMC Med Genet. 2011 Jan 19;12:14. doi: 10.1186/1471-2350-12-14 (PMC3037298; doi:10.1186/1471-2350-12-14)
Supplement: Additional File 3 — Table S3. cDNA samples used for sequencing of PEPD. Case/control status, nationality of origin, sex and family history of each individual whose sample was used in the sequencing of PEPD provided in tabular format. [file 1471-2350-12-14-S3.PDF]

Additional File 3

Table S3. cDNA samples used for sequencing of *PEPD*

Sample	Status	Origin	Sex	Family History
30	Case[1]	Sweden	M	Yes
31	Case	USA	M	Yes
32	Case	USA	M	Yes
33	Case	USA	M	No
34	Case	Canada	M	Yes
35	Case	Canada		Yes
36	Case	Canada		Yes
37	Case	Canada		Yes
38	Case	Canada		Yes
39	Case	Canada		Yes
40	Case	Canada		Yes
41	Case	Canada	M	Yes
42	Case	USA	M	Yes
43	Control	USA	M	No
44	Control	USA	F	No
45	Case	USA	F	Yes
46	Case	USA	M	Yes
47	Case	USA	M	Yes
48	Case	USA	M	Yes
49	Case	Finland	M	Yes
50	Case	USA	M	Yes
51	Case	USA	M	Yes
52	Case	USA	M	Yes

RNA was isolated from cultured skin fibroblasts of AAA cases and controls and reverse transcribed to cDNA for use in PCR amplification (Tromp et al. 1993).

[1]Diagnosis of both AAA and IA.

Tromp G, Wu Y, Prockop DJ, Madhatheri SL, Kleinert C, Earley JJ, Zhuang J, Norrgård Ö, Darling RC, Abbott WM, Cole CW, Jaakkola P, Ryynänen M, Pearce WH, Yao JST, Majamaa K, Smullens SN, Gatalica Z, Ferrell RE, Jimenez SA, Jackson CE, Michels VV, Kaye M, Kuivaniemi H: **Sequencing of cDNA from 50 unrelated patients reveals that mutations in the triple-helical domain of type III procollagen are an infrequent cause of aortic aneurysms.** *J Clin Invest* 1993, 91:2539-2545.
