# Supplementary material for: Analysis of positional candidate genes in the AAA1 susceptibility locus for abdominal aortic aneurysms on chromosome 19
Source: BMC Med Genet. 2011 Jan 19;12:14. doi: 10.1186/1471-2350-12-14 (PMC3037298; doi:10.1186/1471-2350-12-14)
Supplement: Additional File 4 — Table S4. PCR primer pairs for CEPBG, PEPD and CD22. For each PCR reaction, a description of each reaction, primer pair sequences and orientation, product sizes, annealing temperatures, and the cycling times and magnesium chloride concentrations used provided in tabular format. [file 1471-2350-12-14-S4.PDF]

Additional File 4

Table S4. PCR primer pairs for *CEPBG*, *PEPD* and *CD22*.

Reaction	Sequence	Product Size (bp)	Annealing Temp (°C)	Cycling Times (Seconds)	MgCl (mM)
CEBPG Coding	F: CGTGGAAACCATTGATCACCCT<br>R: AGCCTCCAATGGGTCTTTGAGTC	640	57	30-30-60	1.5
PEPD cDNA #1	F: CATGCATTGGCACCCGGAG<br>R: CTTGCTGATGCCGTCAAAGGAG	569	54	60-45-60	1.5
PEPD cDNA #2	F: TCCATTCCAAGGAGCACTTCAAGG<br>R: AAAGGAGCAGGTGATGTCGG	544	54	60-45-60	1.5
PEPD cDNA #3	F: TGGAAAGCCTCTTCGAGCACTA<br>R: GATTAAAGGTGGGAGCCTGCAAA	1022	54	60-45-60	2.0
CD22 Exon 1	F: AACCCATACACGGAAAGGAAGAAC<br>R: GGCATCCAATAACCTACCCTACTC	472	56	60-45-60	1.5
CD22 Exon 2	F: GCCAGAGAAACGCTTGAGC<br>R: CAGAGATACACAAAGACAGCAGGA	527	56	60-45-60	1.5
CD22 Exon 3	F: TGGACAACATAGCAAGACC<br>R: TGGTGAGAAAGAAAGAGGGAAAG	819	58	60-45-60	1.5
CD22 Exon 4	F: GACTGGAGAGAGGTGGAATGAAG<br>R: TGAATGAATGAGGCACAAGATAATGATG	732	58	60-45-60	1.5
CD22 Exons 5-6	F: AGAAGTAGAGGAAGTGGGAGG<br>R: CATCTGCCTTCCCCGTTC	915	58	60-45-60	1.5
CD22 Exons 7-8	F: GCACCAAACCACAGCCAG<br>R: GCACTCTTCCCCTCCATCAC	1086	58	60-45-60	1.5
CD22 Exons 8-9	F: GCTGGGATGAGGGGATGAAG<br>R: AGGCTGAGGCGAAAGGATTG	1110	58	60-45-60	1.5
CD22 Exons 10-11	F: CCGTTGTCACCCTCTTCCTC<br>R: ATGTTGGCTACGCTGGTCTC	558	58	60-45-60	1.5
CD22 Exon 12	F: GGGAAACAAGGTGAAGGAAGG<br>R: GAGTGGGAATGAGGCAGAG	372	56	60-45-60	1.5
CD22 Exon 13	F: CATAAGGGGCTGAAGTTGG<br>R: GAGTTTTGGAGGTTTGGGGTTAC	866	58	60-45-60	1.5
CD22 Exon 14 #1	F: GGGTGGAATGAAGGAGAGAATG<br>R: CAGGAGGGACACAAGCAG	564	54	60-45-60	1.5
CD22 Exon 14 #2	F: GTTCTCTTCCACTCTCCTTGCTAC<br>R: CACTGTGACTCCATTTCCTTCC	537	56	60-45-60	1.5
