# Supplementary material for: Analysis of positional candidate genes in the AAA1 susceptibility locus for abdominal aortic aneurysms on chromosome 19
Source: BMC Med Genet. 2011 Jan 19;12:14. doi: 10.1186/1471-2350-12-14 (PMC3037298; doi:10.1186/1471-2350-12-14)
Supplement: Additional File 5 — Table S5. Sequencing primers used for CEBPG, PEPD and CD22. For each sequencing reaction, a description of the reaction, the primer sequences and orientation, and predicted melting points for primer provided in tabular format. [file 1471-2350-12-14-S5.PDF]

Additional File 5

Table 5. Sequencing primers used for *CEBPG, PEPD* and *CD22*.

PCR Reaction	Primer Sequence	Tm (°C)
CEPBG	F1: CCTGCTCTCATTTCTACCTGTTCT	56.3
Coding	F2: CAACGCCGAGAGAGGAACAA	57.4
	R1: AGCCATTGACATGAGTGGTG	55.3
	R2: GTGTGTCTTGTGCTTTCTGCT	55.9
PEPD	F: GCTGACGCCGCACTTCAC	59.9
cDNA #1	R: CTGCCGCTGTCCGTGTTG	59.6
PEPD	F: TAGATGAGATTGCCAGCGTC	54.5
cDNA #2	R: CCCATGTCGAACAGGCAC	56.4
PEPD	F1: ATGCGCCACAGCTCCTAC	57.9
cDNA #3	F2: TGGGTGCCATGAAGCCAG	58.3
	R1: GCCGTCTCTCGCTACTGG	57.8
	R2: TCCACGCCCTCTGGGTAG	59.1
CD22	F: TCAGAGCCATAGAGAAGCAGG	56.1
Exon 1	R: GGTAAGAGAGACCAGGGAG	56.4
CD22	F: CATCCCAAATGCCACATCC	54.2
Exon 2	R: CTGGGTCTTTCCTCCTCCTC	56.3
CD22	F: TGGACAACATAGCAAGACC	52.2
Exon 3	R: GCCATCGTCATCCGCTG	56.6
CD22	F: GGTGATTTGGGACAGGACA	54.7
Exon 4	R: GAAACACCAGGGCTGACAAG	56
CD22	F: GCACTTTCCACACCCTCC	56.2
Exons 5-6	R: CATCTGCCTTCCCCGTTC	56
CD22	F: ACAGGTAGCGGGAGAAGAG	56
Exons 7-8	R: CACTGGTCCCTTCTCTGCTC	57.4
CD22	F: GGACAGCAAAAGGGACAGG	56.1
Exons 8-9	R: CAGTGAGGTGTCATTATGCGAC	55.8
CD22	F: AGAAGGACGAGTCTGGCTG	56.5
Exons 10-11	R: ATGTTGGCTACGCTGGTCTC	57.3
CD22	F: GGTGAAGGAAGGGGATAAAATGTC	55.6
Exon 12	R: TGGCACAAAGAGGGCAGG	58.2
CD22	F: TGTTGAGAGGGAGGAGAGTTC	55.7
Exon 13	R: TCATAGTCGCCCTGAGAAAACC	56.7
CD22	F: GGGTGGAATGAAGGAGAGAATG	55.3
Exon 14 #1	R: CAGGAGGGACACAAGCAG	55.6
CD22	F: CCATCTAAATACCTGCCCTGAC	55.1
Exon 14 #2	R: AGACTTTCCCCTCTCCTCATC	55.6

Tm, Predicted melting point
