# Supplementary material for: Analysis of positional candidate genes in the AAA1 susceptibility locus for abdominal aortic aneurysms on chromosome 19
Source: BMC Med Genet. 2011 Jan 19;12:14. doi: 10.1186/1471-2350-12-14 (PMC3037298; doi:10.1186/1471-2350-12-14)
Supplement: Additional File 6 — Table S6. Human tissue samples used in immunohistochemical analysis of CD22 and PEPD. Donor age, sex, case/control status and control cause of death if known. [file 1471-2350-12-14-S6.PDF]

Additional File 6

Table S6. Human tissue samples used in immunohistochemical analysis of *CD22* and *PEPD*

ID	Age (years)	Sex	Diagnosis	Cause of Death
WSU037	79	M	AAA	NA
WSU038	67	M	AAA	NA
WSU039	64	M	AAA	NA
WSU048	87	F	AAA	NA
WSU050	87		AAA	NA
WSU052	70	M	AAA	NA
WSU064	63	M	AAA	NA
WSU068	72	M	AAA	NA
WSU075	67	M	AAA	NA
JL6183	88	F	Control	Trauma
JL6074	69	F	Control	Head trauma due to fall
A07-83	87	F	Control	
A07-84	58	M	Control	
A07-87	62	M	Control	
A07-88	58	F	Control	
A07-90	68	M	Control	

AAA samples were obtained as part of elective repair operations and would have been otherwise discarded. Control samples were obtained at autopsy. All individuals were Caucasian.
