# Supplementary material for: Analysis of positional candidate genes in the AAA1 susceptibility locus for abdominal aortic aneurysms on chromosome 19
Source: BMC Med Genet. 2011 Jan 19;12:14. doi: 10.1186/1471-2350-12-14 (PMC3037298; doi:10.1186/1471-2350-12-14)
Supplement: Additional File 7 — Figure S1. Linkage disequilibrium (LD) plots of genotyped SNPs in CEBPG and PEPD for cases (A) and controls (B) separately. LD at the CEBPG/PEPD locus plotted separately for cases and controls using r2 as the statistic. Approximate locations of genes and SNPs were plotted along the x-axis above plots. Nominally associated SNPs are indicated with an asterisk. [file 1471-2350-12-14-S7.PDF]

Additional File 7.

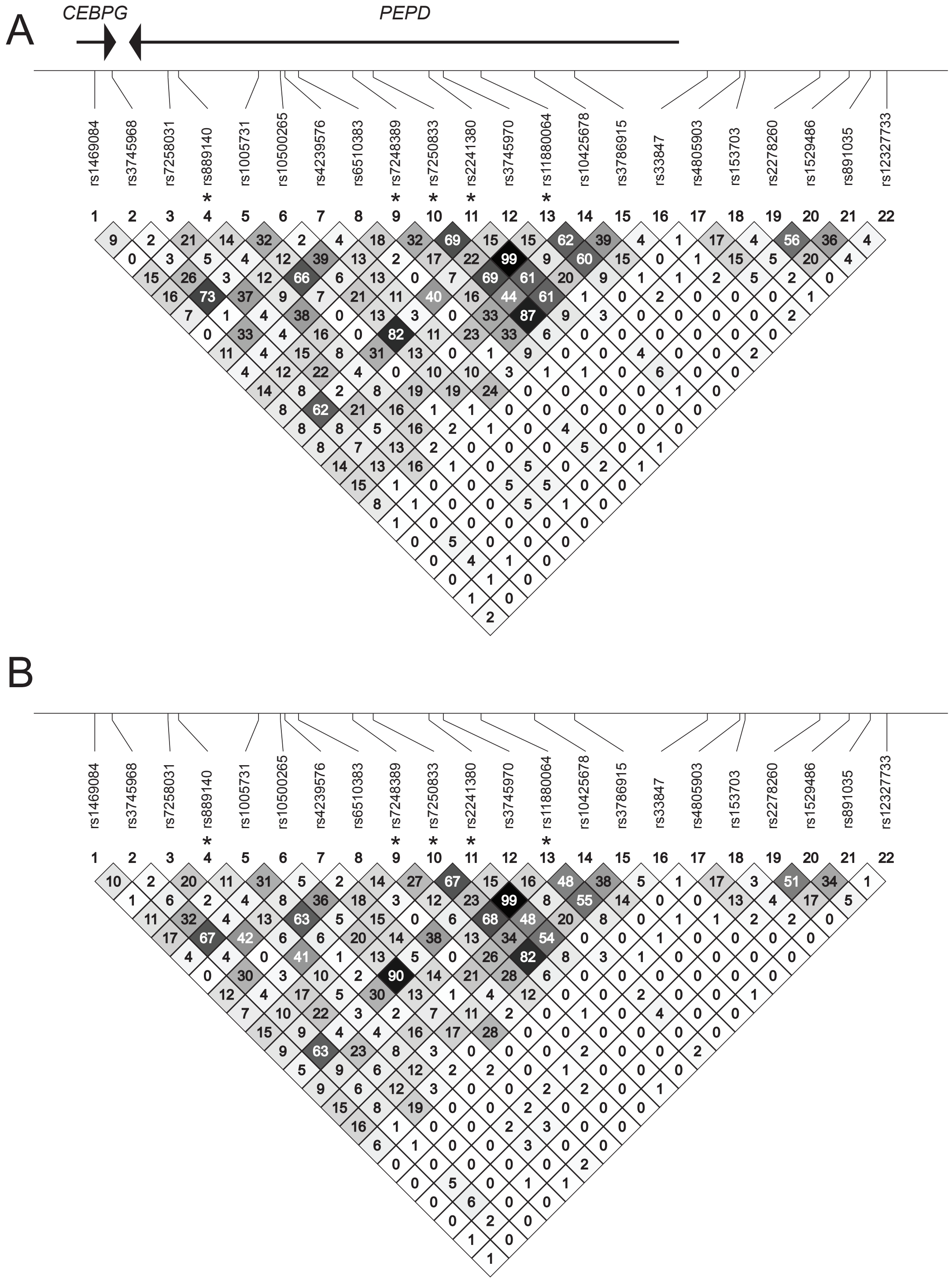

Figure S1. Linkage disequilibrium (LD) plots of genotyped SNPs in CEBPG and PEPD for cases (A) and controls (B) separately. Case (A) and control (B) genotype frequency data for each SNP genotyped in the region of CEBPG and PEPD were used to generate the plots in Haploview software. LD plots were constructed using r^2 as the LD statistic, with black indicating complete LD and white indicating no LD. SNPs marked with an asterisk (*) were nominally associated with AAA. For details on SNPs, see Additional file 1, Table S1.
