# Supplementary material for: Analysis of positional candidate genes in the AAA1 susceptibility locus for abdominal aortic aneurysms on chromosome 19
Source: BMC Med Genet. 2011 Jan 19;12:14. doi: 10.1186/1471-2350-12-14 (PMC3037298; doi:10.1186/1471-2350-12-14)
Supplement: Additional File 8 — Figure S2. LD plots of genotyped SNPs in CD22 for cases (A) and controls (B) separately. LD at the CD22 locus plotted separately for cases and controls using r2 as the statistic. Approximate location of CD22 and SNPs were plotted along the x-axis above plots. Nominally associated SNPs are indicated with an asterisk. [file 1471-2350-12-14-S8.PDF]

Additional File 8.

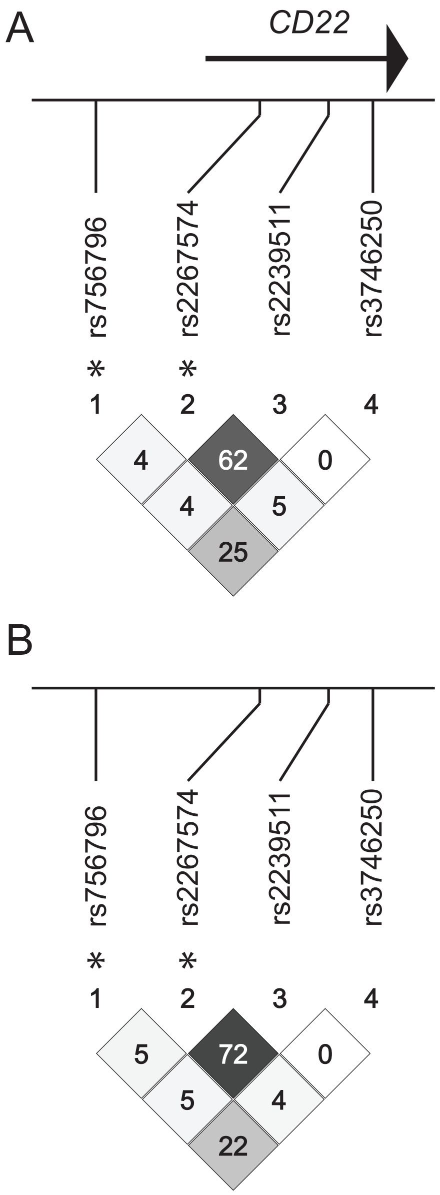

Figure S2. LD plots of genotyped SNPs in CD22 for cases (A) and controls (B) separately. LD plots were constructed in a manner similar to that described in Additional file 7, Figure S1. SNPs marked with an asterisk (*) were nominally associated with AAA. For details on SNPs, see Additional file 1, Table S1.
