# Supplementary material for: Analysis of positional candidate genes in the AAA1 susceptibility locus for abdominal aortic aneurysms on chromosome 19
Source: BMC Med Genet. 2011 Jan 19;12:14. doi: 10.1186/1471-2350-12-14 (PMC3037298; doi:10.1186/1471-2350-12-14)
Supplement: Additional File 9 — Table S7. PEPD sequence changes by sample number. Table showing genotype at each sequence variant detected by sequencing in each individual sequenced. Sample numbers refer to Additional file 3, Table S3, and sequence change number refers to Table 4. [file 1471-2350-12-14-S9.PDF]

Additional File 9

Table S7. *PEPD* sequence changes by sample number.

	Sequence Change[1]						
**Sample[2]**	**1**	**2**	**3**	**4**	**5**	**6**	**7**
30	T/T	T/T	C/T	C/C	G/G	C/T	G/G
31	T/T	T/T	C/C	C/C	G/G	C/C	G/G
32	T/T	T/T	C/C	C/C	G/G	C/T	G/G
33	T/T	T/T	C/C	C/C	G/G	C/C	G/G
34	T/T	T/T	C/C	C/T	G/G	C/C	G/G
35	T/C	T/T	C/C	C/C	G/G	C/C	G/G
36	T/T	T/T	C/C	C/C	G/G	T/T	G/G
37	T/C	T/T	C/C	C/C	G/G	C/C	G/G
38	T/T	T/T	C/C	C/C	G/G	C/T	G/G
39	T/T	T/T	C/C	C/C	G/G	C/C	G/G
40	T/T	T/T	C/C	C/C	G/G	T/T	G/G
41	T/T	T/T	C/C	C/T	G/G	C/C	G/G
42	T/T	T/T	C/C	C/T	G/G	C/C	G/G
43	T/T	T/T	C/C	C/T	G/G	C/T	G/G
44	T/T	T/T	C/C	C/C	G/G	C/C	G/G
45	T/T	T/T	C/C	C/C	G/G	C/T	G/G
46	T/T	T/T	C/C	C/C	G/G	C/C	G/T
47	T/T	T/T	C/C	C/C	G/G	C/C	G/G
48	T/T	T/T	C/C	C/C	G/G	C/T	G/G
49	T/C	T/C	C/C	C/C	G/G	C/C	G/G
50	T/T	T/T	C/C	C/C	G/G	C/T	G/G
51	T/T	T/T	C/C	C/T	G/A	C/C	G/G
52	T/T	T/T	C/C	C/C	G/G	C/C	G/G

[1]Sequence Change indicates the genotype at the corresponding position from Table 4 in the main manuscript for each individual whose DNA was sequenced.
[2]Sample number refers to samples described in Additional file 3, Table S3. Samples 43 and 44 were from controls.
Highlighted boxes indicate the presence of at least one minor allele in that individual.
