# Supplementary material for: Analysis of positional candidate genes in the AAA1 susceptibility locus for abdominal aortic aneurysms on chromosome 19
Source: BMC Med Genet. 2011 Jan 19;12:14. doi: 10.1186/1471-2350-12-14 (PMC3037298; doi:10.1186/1471-2350-12-14)
Supplement: Additional File 10 — Table S8. Flanking sequences of the PEPD sequence variants. Gene feature, 15 bp flanking sequences and SNP identifier, if available, for each sequence variant. [file 1471-2350-12-14-S10.PDF]

Additional File 10

Table S8. Flanking sequences of the *PEPD* sequence variants.

**Sequence Change[1]**	**Feature**	**Flanking Sequence**	**SNP Identifier**
1	Exon 9	GGGAATGAAAGAATA (T/C) GAGTTGGAAAGGTAA	rs3745969
2	Exon 11	GGCTCTTTGCAGTGG (T/C) GAGAACTCAGCCGTG	rs74988985
3	Exon 13	CGTGTTTATGCCTCA (C/T) GGGCTTGGCCACTTC	
4	Exon 13	GGGCATTGACGTGCA (C/T) GACGTGGGAGGCTAC	rs17569
5	Exon 14	GCGGACCCGGCCCGC (G/A) CCTCCTTCCTTAACC	
6	Exon 14	GCCCGCGCCTCCTTC (C/T) TTAACCGCGAGGTCC	rs17570
7	Exon 15	CAGCGGCATAGAGCT (G/T) CTGACCTGCGTGCCC	

[1]The numbers in this column refer to the numbering used in Additional file 9, Table S7 and Table 4 in the main manuscript.
