# Supplementary material for: Analysis of positional candidate genes in the AAA1 susceptibility locus for abdominal aortic aneurysms on chromosome 19
Source: BMC Med Genet. 2011 Jan 19;12:14. doi: 10.1186/1471-2350-12-14 (PMC3037298; doi:10.1186/1471-2350-12-14)
Supplement: Additional File 12 — Table S10. Flanking sequences of the CD22 sequence variants. Gene feature, 15 bp flanking sequences and SNP identifier, if available, for each sequence variant. [file 1471-2350-12-14-S12.PDF]

Additional File 12.

Table S10. Flanking sequences of the *CD22* sequence variants.

Sequence Change[1]	Feature	Flanking Sequence	SNP Identifier
1	Intron 3	AGAGCTGCGGGACCT (C/T) GGATGTCCCATCTGA	rs881456
2	Intron 3	GTCCCATCTGACCCT (C/G) AGTTCCTGCCCCTCT	rs10419538
3	Intron 6	AGAGAAGATGGACAC (A/G) GGGAACGGGGAAGGC	rs7248108
4	Exon 7	CACAACCATCGCCTG (C/T) GCAGCTTGTAATAGT	rs25677
5	Exon 8	GGGAGAAAAATGGCA (G/A) GCTTCTGGGGAAAGA	
6	Intron 9	AGGCCTCCTTCCTGC (T/A) CTTGTTCTTCTTGGT	
7	Intron 10	ACCCTCACACATGTG (C/T) CTTTATTTCTCAGTT	rs45453699
8	Exon 12	CCCTCCCAGGTTAGA (A/G) GGGCCCCCCTCTCTG	
9	Exon 12	CCCCCCTCTCTGAAG (G/A) CCCCCACTCCCTGGG	rs10406069
10	Exon 12	CACCCTGCGCTTTCC (C/A) GAGATGAACATACCA	rs34826052
11	Intron 13	CAAGTGGTAAGGAGG (G/A) TCTCCCCAGGTCTCC	hCV25603572
12	Intron 13	AGTGGTAAGGAGGGT (C/T) TCCCCAGGTCTCCCC	
13	Intron 13	CCAGGTCCTGGATGC (C/T) GGCCACAGCCAGTTT	rs58156121
14	Intron 13	CGGCGGTGGAGGCTG (G/A) CGACAGTGGGGCCAG	rs12985354
15	Exon 14	TGGCTTCCTCCTGCG (C/T) GCATGTGCGCACACA	rs73031792
16	Exon 14	TGCGCGCATGTGCGC (A/G) CACACACACACACAC	rs35529786
17	Exon 14	CACACACACACGCAC (-/GC) ACACACACACACACA	rs34472317
18	Exon 14	CAAACCTCCAAAACT (C/T) CTGCCCCTGTTCTCT	
19	Exon 14	CACTCTCCTTGCTAC (C/T) CAGAAATCCATCTAA	rs16970255
20	Exon 14	AGAAAAGGACAGAAA (C/T) GAAGTAGAAAGGGGC	rs3088063

[1]The numbers in this column refer to the numbering used in Additional file 11, Table S9, and Table 5 in the main manuscript.
